# Dynamic metabolic patterns tracking neurodegeneration and gliosis following 26S proteasome dysfunction in mouse forebrain neurons

**DOI:** 10.1038/s41598-018-23155-2

**Published:** 2018-03-19

**Authors:** Philippine C. Geiszler, Aslihan Ugun-Klusek, Karen Lawler, Marie-Christine Pardon, Ding Yuchun, Li Bai, Clare A. Daykin, Dorothee P. Auer, Lynn Bedford

**Affiliations:** 1 Division of Clinical Neuroscience, School of Medicine, University of Nottingham, Nottingham, UK; 2 School of Pharmacy, University of Nottingham, Nottingham, UK; 3 School of Science and Technology, Nottingham Trent University, Nottingham, UK; 4 School of Life Sciences, University of Nottingham, Nottingham, UK; 5 School of Computing, University of Newcastle, Newcastle, UK; 6 School of Computer Sciences, University of Nottingham, Nottingham, UK; 7 Metaboconsult UK, Heanor, Derbyshire UK; 8 Sir Peter Mansfield Imaging Centre, School of Medicine, University of Nottingham, Nottingham, UK

## Abstract

Metabolite profiling is an important tool that may better capture the multiple features of neurodegeneration. With the considerable parallels between mouse and human metabolism, the use of metabolomics in mouse models with neurodegenerative pathology provides mechanistic insight and ready translation into aspects of human disease. Using 400 MHz nuclear magnetic resonance spectroscopy we have carried out a temporal region-specific investigation of the metabolome of neuron-specific 26S proteasome knockout mice characterised by progressive neurodegeneration and Lewy-like inclusion formation in the forebrain. An early significant decrease in *N*-acetyl aspartate revealed evidence of neuronal dysfunction before cell death that may be associated with changes in brain neuroenergetics, underpinning the use of this metabolite to track neuronal health. Importantly, we show early and extensive activation of astrocytes and microglia in response to targeted neuronal dysfunction in this context, but only late changes in myo-inositol; the best established glial cell marker in magnetic resonance spectroscopy studies, supporting recent evidence that additional early neuroinflammatory markers are needed. Our results extend the limited understanding of metabolite changes associated with gliosis and provide evidence that changes in glutamate homeostasis and lactate may correlate with astrocyte activation and have biomarker potential for tracking neuroinflammation.

## Introduction

Neurodegenerative diseases such as Alzheimer’s disease (AD) are complex and the underlying mechanisms involved unclear. Studies of neurodegeneration need to temporally capture multiple molecular entities and their interactions to advance our knowledge of early disease mechanisms and identify biomarkers that represent pathological processes at a molecular or cellular level. Metabolomics is an important systems approach that can identify changes in numerous individual metabolites representing a multitude of functional pathways that may better characterise the multiple features of neurodegenerative disease and lead to the development of effective therapeutic strategies^1–3^.

Current paradigms for the cause and progression of major human neurodegenerative diseases indicate that abnormal protein aggregation, impaired protein degradation, mitochondrial dysfunction, oxidative stress and neuroinflammation are involved in various ways. The ubiquitin proteasome system (UPS); the major pathway for the degradation of damaged, misfolded and aggregation-prone proteins, has been linked to neurodegenerative disease since ubiquitin was found in their characteristic protein aggregates^4–8^. Proteasome activity declines during aging and UPS dysfunction is supported by recent genome-wide association studies in AD^9,10^. Further, a direct role for the UPS in the pathogenic mechanisms of neurodegenerative disease was demonstrated by inactivation of the 26S proteasome in mouse neurons, causing neurodegeneration and Lewy-like inclusions resembling human pale bodies^11^. Autophagy, the other major protein quality control pathway in the cell, was also linked to neurodegenerative disease following the observation that loss of autophagy in mouse neurons causes accumulation of ubiquitin-positive aggregates and neurodegeneration, however, a recent study suggests ubiquitinated proteins are not major targets for basal autophagy^12,13^. Mitochondrial dysfunction, particularly altered oxidative phosphorylation, has been convincingly associated with disease pathogenesis, and disease-linked proteins shown to have a detrimental effect on mitochondrial function and increase reactive oxygen species production^14–17^. Neuroinflammation is increasingly being recognised, not only as a pathological hallmark, but as a contributor to neurodegenerative disease, characterised by the reactive morphology of astrocytes and microglia, and release of inflammatory mediators^18–26^. With the considerable parallels between rodent and human metabolism, the metabolic profiles identified in animal models with neurodegenerative pathology directly provide mechanistic insight and ready translation to some aspects of human disease.

*N*-acetyl aspartate (NAA) as one of the most prominent metabolites in ^1^H magnetic resonance spectroscopy (*MRS*) of the brain is employed as a general indicator of neuronal health, and specifically mitochondrial function^27–31^. Clinical studies and experimental models show NAA reduction correlates with neuronal dysfunction and neurodegeneration in various neurodegenerative conditions, including Alzheimer’s and Parkinson’s diseases^32–35^. Our understanding of which metabolite(s) best track neuroinflammation *in vivo*, however, is limited^18–21^. Myo-inositol has been shown to be a glial cell marker with consistent clinical evidence of elevated myo-inositol in a range of diseases characterised by astrogliosis^36–42^. The inferential notion that myo-inositol levels detected by *in vivo* ^1^H *MRS* reflect the extent of activation of astrocytes and microglia is less well supported. Recent data has been controversial and activation of glia has been associated with glutamate/glutamine, lactate, choline-containing compounds as well as macromolecules and lipids^36–42^.

Using 400 mHz nuclear magnetic resonance (*NMR*) spectroscopy we have carried out a temporal region-specific characterisation of the metabolome of neuron-specific 26S proteasome knockout mice (*Psmc1*^fl/fl^;*CaMKIIα-Cre*) between 2 and 6 weeks-old accompanying progressive neurodegeneration and Lewy-like inclusion formation in the forebrain. Importantly, we have combined our *NMR* spectroscopy analysis with quantitative histopathological and molecular investigations of activated astrocytes and microglia in *Psmc1*^fl/fl^;*CaMKIIα-Cre* mouse forebrain to inform on putative metabolite changes that may track gliosis.

## Materials and Methods

### Mice

*Psmc1*^fl/fl^;*CaMKIIα-Cre* and control (*Psmc1*^fl/fl^;*CaMKII*α*-Wt* or *Psmc1*^fl/wt^;*CaMKII*α*-Wt)* littermate mice were housed under identical conditions and genotyped as described previously^11^. Mice had ad libitum access to food and water. Female mice were used in all analyses. Cortex (50 mg), hippocampus (10 mg) and cerebellum (30 mg) were rapidly micro-dissected from mouse brain, snap-frozen in liquid nitrogen and transferred directly to −80 °C for storage until extraction. Samples were collected at the same time on each day over a period of 4 months before tissue extraction for ^1^H NMR Spectroscopy. The number of mice used at each age in NMR spectroscopy was determined by power calculations using online software (https://www.stat.ubc.ca/~rollin/stats/ssize/n2.html) based on unpublished pilot data of n = 3 mice at each age (power 0.80; alpha 0.05). Actual number of mice used at each age met or exceeded power calculations and is provided in Supplementary Table 1. All procedures were authorised and approved by the University of Nottingham ethics committee and carried out in accordance with the UK Animals (Scientific Procedures) Act 1986.

### Tissue Extraction for ^1^H NMR Spectroscopy

Frozen tissues (Supplementary Table 1) were extracted using a previously recommended chloroform-methanol procedure^43^. Samples were first homogenised in water using a ball mill (2 min, 30/s; Retsch MM 301; Retsch GmbH, Germany). The homogenates were further treated with chloroform, methanol and additional water; 8 mL solvent per mg tissue at a final solvent ratio of 2:2:3 (chloroform:methanol:water)^44^. After the two liquid extract layers were separated, the solvents were removed using a concentrating evaporator system without heating (Jouan RC 10.22; Jouan, France). The hydrophilic residues were reconstituted in phosphate-buffered (pH 7.2; 0.1 M) deuterated water containing 3-(trimethylsilyl)-2,2′,3,3′-tetradeuteropropionic acid (TSP; 0.334 nmol/ml) as reference substance prior to their spectroscopic analysis.

### ^1^H NMR Spectroscopy

Liquid state NMR spectroscopy was performed using a Bruker Avance spectrometer (Bruker BioSpin GmbH, Germany) equipped with a 5 mm SEL probe (1 H/D XYZ-gradient probe) operating at a proton frequency of 400.13 MHz. All one-dimensional spectra were recorded at 298.2 K without spinning using a conventional water presaturation pulse sequence based on the first increment of a nuclear Overhauser effect spectroscopy sequence (32 k data points, spectral width = 13 ppm, number of scans = 128/sample, relaxation delay = 2 s, mixing time = 0.12 s, acquisition time = 3.15 s).

The TopSpin software (v. 2.1; Bruker BioSpin GmbH, Germany) was used for spectral processing whereby the free induction decays (FIDs) were zero-filled to twice the number of data points and apodised by an exponential weighing function, applying a line-broadening factor of 0.3 Hz. All spectra were Fourier transformed, phased, baseline corrected and calibrated to the TSP reference signal at δ 0.00 ppm.

### Spectrum Processing

To prepare the spectral data for pattern recognition analysis, the data points ranging from 9.46 to 0.86 ppm, exclusive of solvent signals, were collected into successive 0.04 ppm wide buckets using the AMIX software (v. 3.8; Bruker BioSpin GmbH, Germany). To minimise any statistical contribution of tissue mass, all spectra were normalised to the total spectral area, *i*.*e*. the sum of all included data points.

Relative quantification of individual metabolites was performed by the sum of their data points in relation to the total spectral area, as calculated by the AMIX software. Metabolite quantification was limited to a set of 30 specific metabolites. Peak annotations, some of which are denoted in Supplementary Fig. 1, were informed by the literature^3,45,46^, in-house and web-based data bases^47,48^, as well as two-dimensional NMR spectra, such as total correlation (TOCSY) and j-resolved spectroscopy (jRES).

### Multivariate Statistical Analysis

Data pre-treatment and pattern recognition analysis were performed using SIMCA-P (v. 11.0.0.0; Umetrics AB, Sweden). The bucketed dataset was pre-treated by mean-centring with and without scaling to unit variance in order to separately investigate the contribution of small and large signals to the statistical models.

Principal component analysis (PCA) was performed to obtain information about major trends within datasets^49^. PCA is a method to reduce the complexity of multidimensional datasets, such as spectra, to a few latent variables termed principal components (PCs). Projections of the original spectral data onto the newly calculated PCs (‘scores plots’) allow the observation of sample clusters illustrating spectral similarities between samples. The corresponding ‘loadings plots’ enable the metabolic interpretation of such clusters^49^. The PCA results were assessed through the parameter R^2^X*(cum)* describing the amount of spectral data variation explained by the PCs.

Projection to latent structures by partial least squares (PLS) regression and discriminant analysis (PLS-DA) were deployed to define distinct group clusters relating to animal age and genotype, respectively. The spectral data (so-called ‘X’-block) is related to the ‘Y’-block, which constitutes of numerical data (*e*.*g*. age) in the case of PLS regression analysis or two categorical groups (*e*.*g*. *Psmc1*^fl/fl^*;CaMKIIα-Cre* and control) in the case of PLS-DA, in order to inform about the robustness of their relationship as well as X’s predictability of Y. For further details on the PLS regression and PLS-DA as well as validation see supplementary experimental information.

### Univariate Statistical Analysis

Similar to previous studies, Kolmogorov-Smirnov tests demonstrated that the endogenous metabolites in our study did not show Gaussian distribution^50^. Therefore, Mann-Whitney-U tests and the Z-statistic^51^ were applied to compare individual metabolite concentrations between the two genotypes. Spearman correlation coefficients *r*_*μ*_ and *r*_*μμ*_ signified the relationship between metabolite concentration and age or another metabolite concentration, respectively. To minimise the risk of spurious positive results, Bonferroni-corrected *p* values were used.

### Immunoblotting

Mouse cortices were homogenised in lysis buffer [50 mM Tris, 150 mM NaCl, 2 mM EDTA, 1 mM MgCl_2_, 100 mM NaF, 10% glycerol, 1% triton X-100, 1% Na deoxycholate, 0.1% SDS, 125 mM sucrose and 1% (v/v) protease inhibitor cocktail] using a bead beater (MP bio FastPrep®-24). Samples were centrifuged at 10 000 g for 10 minutes at 4 °C. Subcellular fractionation of mouse cortices (100 mg) was performed on ice as described previously^52^. Freshly dissected cortices were homogenised in buffered sucrose solution [2 mM HEPES pH 7.4, 210 mM mannitol, 70 mM sucrose, 0.1 mM EDTA, 1% (v/v) protease inhibitor cocktail] using a Dounce tissue grinder to produce total homogenates. Total homogenates were centrifuged at 500 g followed by 1000 g for 10 min each and the resulting pellet termed the nuclear-enriched fraction. Supernatants were centrifuged at 7000 g for 10 min to produce the cytosolic fraction. Following washing in buffered sucrose solution the pellet was re-suspended and layered onto a 60, 32, 23 and 15% sucrose gradient in 10 mM MOPS (pH 7.2), 1 mM EDTA. Gradients were centrifuged at 130 000 g for 60 min and mitochondria were collected from the 32–60% interface. Mitochondria were pelleted by centrifugation at 10 000 g for 10 min and washed twice with buffered sucrose solution before re-suspending in buffered sucrose solution for storage; all fractions were stored at −80 °C. Protein concentration was determined using Bradford protein assay.

Equal protein was subjected to sodium dodecyl sulfate-polyacrylamide gel electrophoresis followed by transfer to nitrocellulose membrane. Membranes were blocked in 5% dried skimmed milk in Tris-buffered saline (TBS) containing 0.1% (v/v) Tween-20 (TBS-T) for 1 h and then incubated overnight at 4 °C with glial fibrillary acidic protein (1:4000; Sigma G3893), glutamate dehydrogenase 1 (1:1000; VWR 89379–724), glycogen phosphorylase (brain form) (1:1000; GeneTex GTX104291), pyruvate dehydrogenase E1 component subunit alpha (1:1000; Abcam ab110334), neuron-specific nuclear protein (1:1000; Abcam ab177487) or glyceraldehyde 3-phosphate dehydrogenase (1:20 000; Sigma G9545) antibodies in blocking solution. Membranes were washed and incubated for 1 h at room temperature with appropriate LI-COR IRDye® 800CW secondary antibodies in TBS (LI-COR 926-32210 and 926-32211). Bands were visualised using the Odyssey infrared imaging system at 800 nm and intensity quantified using the Odyssey Image Studio (LI-COR Biosciences).

### Immunohistochemistry

Mice were perfusion-fixed with 0.9% saline followed by 4% paraformaldehyde in phosphate buffered saline (pH 7.4). Fixed brains were processed to paraffin and 7 µm horizontal sections cut using a microtome. Immunostaining of ionized calcium-binding adapter molecule 1 (1:200; Wako 019-19741), glial fibrillary acidic protein (1:200; Sigma G3893), cytochrome c oxidase (1:200; Cell Signalling 4844), glutamate dehydrogenase 1 (1:100; GeneTex GTX105765) and glycogen phosphorylase (brain form) (1:60; GeneTex GTX104291) was performed as directed in Vector Laboratories M.O.M Immunodetection (PK2200) or Vectastain Elite Rabbit IgG ABC kits (PK6101) using 0.01 M citrate buffer containing 0.05% (v/v) Tween-20 (pH 6) for antigen retrieval. Immunostained slides were scanned using a Nanozoomer Digital slide scanner (Hamamatsu Photonics) at 40 × magnification and images were viewed using NDP.view2 viewing software.

For quantitation of COXIV-positive aggregates in *Psmc1*^fl/fl^;*CaMKIIα-Cre* neurons annotation tools in NDP.view2 viewing software were used to delineate a 1.4 mm^2^ area including all cortical layers as shown in Supplementary Fig. 9. Counting was performed manually in ImageJ.

### Semi-automated analysis of Iba1 immunohistochemistry

This was carried out as described previously^42^. Levelled Iba1-stained slides were scanned using a Nanozoomer Digital slide scanner (Hamamatsu Photonics) at 40 × magnification. Using custom made software programmed in MATLAB a region of interest (ROI) was drawn on the digitised images outlining the cortex and feature recognition performed^53^. Each image was inspected and corrected manually to avoid artifacts. Analysis provided the area occupied by microglia soma and isolated processes as a percentage of the ROI as well as the soma size.

### Statistics

Details of statistical tests and number of biological replicates (n) are provided in the methods, figures and table legends.

## Results

The *Psmc1*^fl/fl^;*CaMKIIα-Cre* neurodegeneration mouse model has been previously described^11^. *Psmc1*, an essential ATPase subunit of the 19 S regulatory particle of the 26S proteasome, is inactivated at approximately 2 weeks-old in mouse forebrain neurons by conditional gene targeting using *CamKIIα*-driven Cre recombinase, causing progressive 26S proteasome dysfunction between 2 and 4 weeks-old^54–57^. An elegant study describing species chronometry suggests that the final important steps in mouse brain development occur 29.7 days post-conception (postnatal day 12; birth day 0)^58^. *CamKIIα* expression is upregulated at approximately 2 weeks-old, allowing normal CNS development before Cre-mediated recombination of *Psmc1* in post-mitotic neurons. We note the limitation that the mice used in this study are juvenile in the biological context of sexual maturity, but we may consider the brain to be developed and analyses will have applicability to similar processes in human disease. *Psmc1*^fl/fl^;*CaMKIIα-Cre* mice exhibit increasing accumulation of ubiquitinated proteins and paranuclear aggregation of dysfunctional mitochondria from 3 weeks-old^52,57^. Here, to extend previous data reflecting progressive synaptic dysfunction and neurodegeneration, and to correlate with our metabolic profiling analysis we have performed temporal neuron-specific nuclear protein (NeuN) immunoblotting analysis between 2 and 6 weeks-old in control and *Psmc1*^fl/fl^;*CaMKIIα-Cre* forebrain; NeuN was not significantly decreased until 5 weeks-old, indicative of progressive neurodegeneration following *Psmc1* inactivation (Supplementary Fig. 2).

### 26S proteasome dysfunction in forebrain neurons causes metabolic alterations

**Figure 1:**
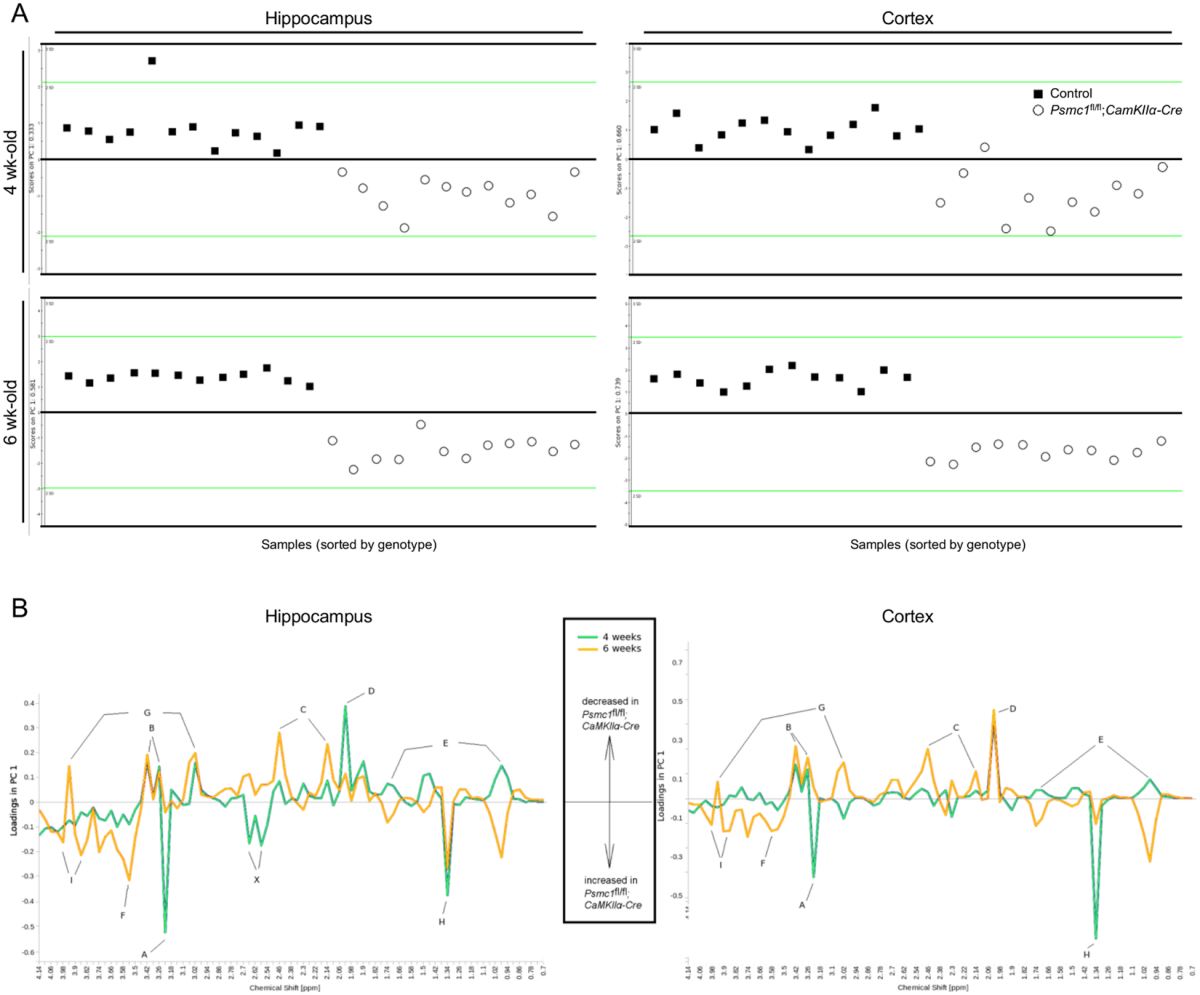
Cortex and hippocampus of *Psmc1*^fl/fl^*;CaMKIIα-Cre* mice display metabolic patterns distinct from controls. (**A**) PCA scores plots illustrating the difference between metabolic profiles of control and *Psmc1*^fl/fl^;*CaMKIIα-Cre* mouse hippocampi and cortices at 4 and 6 weeks-old. (**B**) PCA loadings from 4.14 to 0.7 ppm demonstrating the contribution of individual metabolites to the metabolic profiles of control and *Psmc1*^fl/fl^;*CaMKIIα-Cre* mouse hippocampi and cortices at 4 and 6 weeks-old. Positive and negative loadings indicate a decrease and increase in *Psmc1*^fl/fl^*;CaMKIIα-Cre* mice respectively. A: choline-containing compounds; B: taurine; C: glutamine; D: *N*-acetyl aspartate; E: branch-chained amino acids; F: glycine; G: creatine; H: lactate; I: serine; X: unknown AB system (possibly due to contamination). PCA model figures: R^2^X*(1PC)* = 0.333 (4 week-old) and 0.581 (6 week-old) hippocampus; R^2^X*(1PC)* = 0.660 (4 week-old) and 0.740 (6 week-old) cortex.

**Figure 2:**
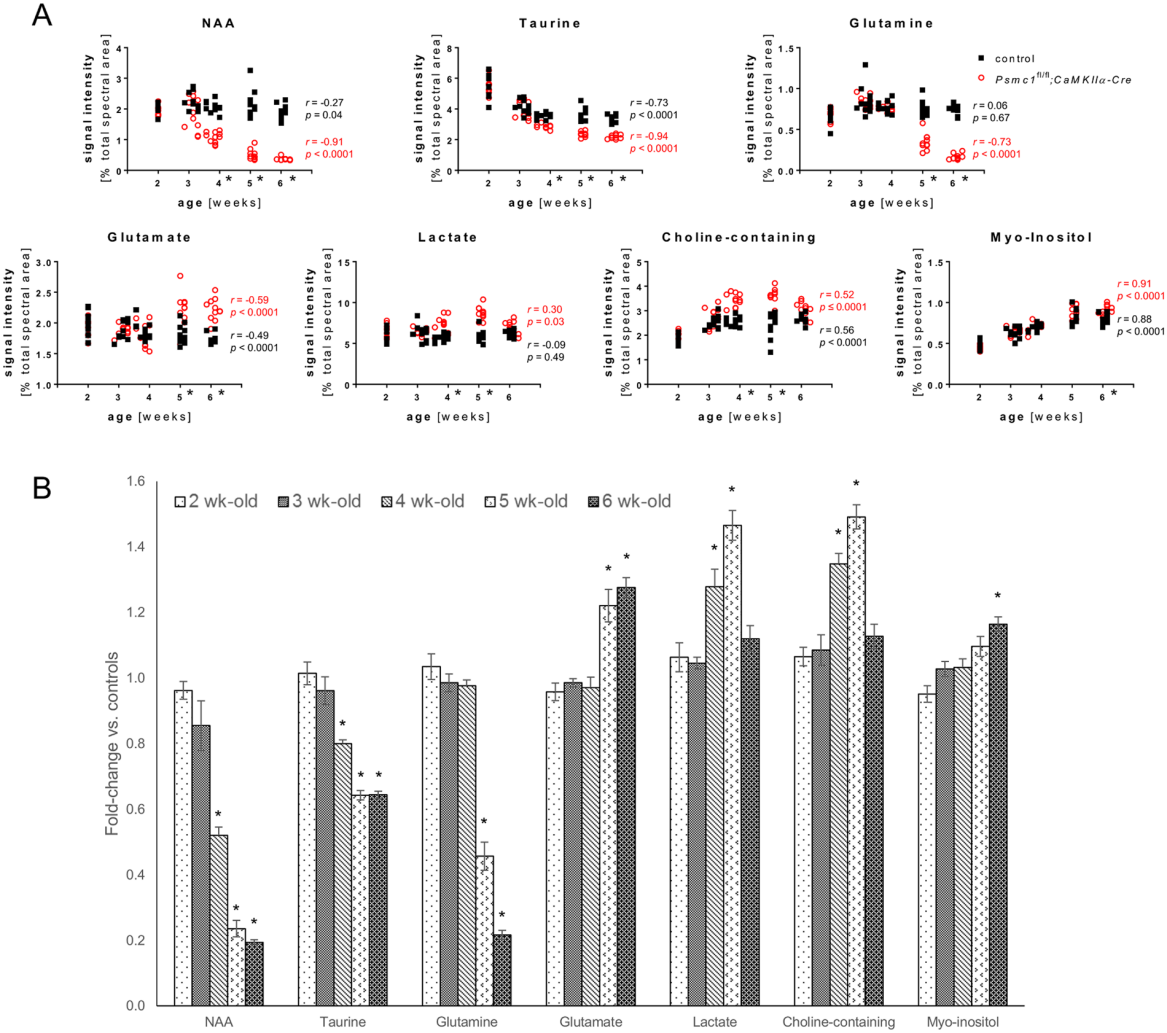
Differences in metabolite concentration between control and *Psmc1*^fl/fl^*;CaMKIIα-Cre* cortices. (**A**) Correlation plots between metabolite concentration and age expressed as % signal intensity relative to the total spectral area for control (black) and *Psmc1*^fl/fl^*;CaMKIIα-Cre* cortices (red). (**B**) Differences in metabolite concentration in *Psmc1*^fl/fl^*;CaMKIIα-Cre* mice expressed as fold-change *vs*. controls. Mean ± SEM. For A and B; n = Supplementary Table 1, *p < 0.002 by Man-Whitney U test.

This study used cortex, hippocampus and cerebellum from control and *Psmc1*^fl/fl^;*CaMKIIα-Cre* mice between 2 and 6 weeks-old. The cerebellum served partly as a control for changes in targeted forebrain neurons, but also to reveal any changes in ‘other’ brain areas that may be secondary to neurodegenerative processes in the forebrain. Multivariate statistical analysis of the whole dataset demonstrated clear metabolic differences between brain regions and age groups in line with previous reports in rodents (Supplementary Fig. 3, Supplementary Tables 2 and 3)^3,46,59,60^. Therefore, genotypic comparisons were obtained separately for each brain region at each age. The cortex and hippocampus of *Psmc1*^fl/fl^*;CaMKIIα-Cre* mice displayed metabolic patterns distinct from controls from 4 weeks-old (Figs 1 and 2, Table 1, Supplementary Fig. 4). Cortex showed the most pronounced metabolic changes and robust multivariate statistical models classified all cortical samples from 4, 5 and 6 week-old mice according to their genotype (Supplementary Table 4). Metabolic changes were confirmed by univariate statistical analysis (Fig. 2 and Table 1). At 4 weeks-old NAA showed the most significant change in *Psmc1*^fl/fl^*;CaMKIIα-Cre* cortices, decreasing to 0.52-fold *vs*. controls, and to 0.24- and 0.19-fold at 5 and 6 weeks-old respectively (Fig. 2 and Table 1). Taurine also showed an early significant decrease in *Psmc1*^fl/fl^*;CaMKIIα-Cre* cortices to 0.8-fold at 4 weeks-old, decreasing to 0.64-fold at 5 and 6 weeks-old (Fig. 2 and Table 1). Early increases in lactate and choline-containing compounds were evident at 4 weeks-old to 1.28- and 1.35-fold respectively, and rose to 1.47- and 1.49-fold at 5 weeks-old (Fig. 2 and Table 1). A significant decrease in glutamine was evident from 5 weeks-old in *Psmc1*^fl/fl^;*CaMKIIα-Cre* cortices to 0.46-fold *vs*. controls, and 0.22-fold at 6 weeks-old; whereas glutamate increased 1.22- and 1.28-fold respectively (Fig. 2 and Table 1). A late 1.16-fold increase in myo-inositol was evident at 6 weeks-old in *Psmc1*^fl/fl^*;CaMKIIα-Cre* cortices (Fig. 2 and Table 1). Except for glutamine, aspartate and alanine, all identified amino acids were increased in *Psmc1*^fl/fl^*;CaMKIIα-Cre* cortices at 6 weeks-old; serine and glycine significantly increased from 5 weeks-old (Supplementary Fig. 5). Corroborative results to the cortex were obtained in the hippocampus, but the cerebellum did not exhibit any significant metabolic differences between *Psmc1*^fl/fl^;*CaMKIIα-Cre* and control mice up to 6 weeks-old, suggesting secondary changes in ‘other’ brain regions are not ‘or not yet’ evident (Table 1, Supplementary Figs 4 and 6).

**Table 1:** Metabolic differences between control and *Pmsc1*^fl/fl^*;CaMKIIα-Cre* mice.

Metabolites	Cortex									Hippocampus									Cerebellum
	4 week			5 week			6 week			4 week			5 week			6 week			6 week
	FC vs. controls	SEM		FC vs. controls	SEM		FC vs. controls	SEM		FC vs. controls	SEM		FC vs. controls	SEM		FC vs. controls	SEM		
Glutamine			—	0.46	0.04	**↓** ^**Z**^	0.22	0.01	↓^Z^	0.85	0.03	↓	0.31	0.05	**↓** ^**Z**^	0.23	0.03	**↓** ^**Z**^	—
NAA	0.52	0.03	**↓** ^**Z**^	0.24	0.03	**↓** ^**Z**^	0.19	0.01	↓^Z^	0.45	0.06	↓	0.44	0.01	↓	0.54	0.02	↓	—
Serine			—	1.24	0.04	**↑**	1.41	0.02	↑^Z^			—	1.27	0.05	↑	1.43	0.07	↑	—
Taurine	0.80	0.01	**↓** ^**Z**^	0.64	0.01	**↓** ^**Z**^	0.64	0.01	↓^Z^			—	0.80	0.03	↓	0.80	0.03	↓	—
Glycerol			—			—	1.11	0.06	↑^Z^			—			—	1.19	0.06	↑	—
Glycine			—	1.18	0.03	↑	1.24	0.02	↑			—	1.27	0.03	↑	1.31	0.04	↑	—
Glutamate			—	1.22	0.05	↑	1.28	0.03	↑			—			—			—	—
Creatine			—			—	0.75	0.02	↓			—			—	0.76	0.02	↓	—
Leucine			—			—	1.43	0.04	↑			—			—	1.26	0.07	↑	—
Phenylalanine			—			—	1.69	0.08	↑			—			—	1.64	0.16	↑	—
Histidine			—			—	1.51	0.04	↑			—			—			—	—
Tyrosine			—			—	1.77	0.08	↑			—			—			—	—
Valine			—			—	1.87	0.09	↑	0.89	0.05	↓			—	1.74	0.15	↑	—
Isoleucine			—			—	1.77	0.08	↑	0.81	0.05	↓			—	1.64	0.12	↑	—
Acetate			—			—	0.77	0.02	↓			—			—	0.63	0.04	↓	—
myo-Inositol			—			—	1.16	0.02	↑			—			—	1.26	0.05	↑	—
Adenosine n.			—			—	0.71	0.02	↓			—			—	0.76	0.03	↓	—
Choline-c.	1.35	0.03	**↑** ^**Z**^	1.49	0.04	↑			—			—			—			—	—
Lactate	1.28	0.05	↑	1.47	0.05	↑			—			—	1.25	0.03	↑			—	—
Aspartate	1.17	0.02	↑			—			—			—			—	0.79	0.02	↓	—
Alanine			—			—			—	0.84	0.02	↓			—			—	—

Differences in metabolite concentration in *Psmc1*^fl/fl^*;CaMKIIα-Cre* mice expressed as fold-change *vs*. controls. Only metabolites that differed between control and *Pmsc1*^fl/fl^*;CaMKIIα-Cre* mice with a *p* < 0.002 by Man-Whitney U test are reported. Arrows indicate the direction of change in *Pmsc1*^fl/fl^*;CaMKIIα-Cre* mice; ^**Z**^ and **bold arrows** signify a positive Z-statistic. n = Supplementary Table 1. NAA, *N*-acetyl aspartate; Choline-c., choline-containing compounds.

### Early glial activation precedes neurodegeneration

**Figure 3:**
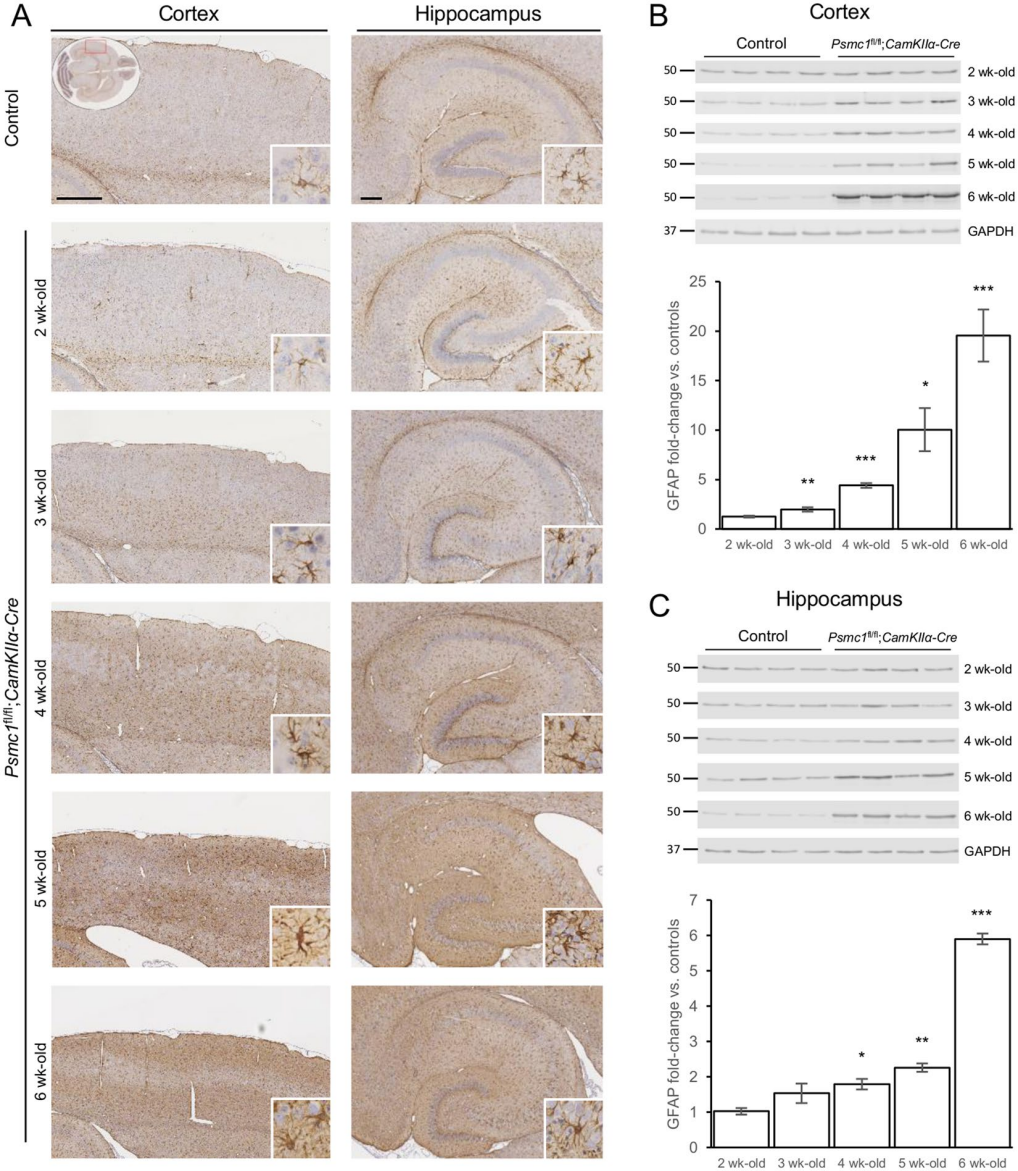
Astrogliosis in *Psmc1*^fl/fl^;*CaMKIIα-Cre* forebrain. (**A**) Representative horizontal brain sections immunostained with glial fibrillary acidic protein (GFAP). No significant differences were evident in control cortices; therefore the control image is representative of all ages. Insets show the region of cortex shown (LHS; oval) and a higher magnification image of the neuropathological finding (RHS; white box). Scale bar = 500 (LHS; cortex) and 200 (RHS; hippocampus) µm. Representative immunoblots and quantification of GFAP in control and *Psmc1*^fl/fl^;*CaMKIIα-Cre* cortices (**B**) and hippocampi (**C**). Glyceraldehyde 3-phosphate dehydrogenase (GAPDH) was used as a loading control at each age for quantification; a representative GAPDH is shown. Images were cropped from those shown in Supplementary Figure 7. Mean ± SEM of n = 4 mice. *p < 0.05, **p < 0.01 and ***p < 0.001 by unpaired Students *t*-test.

Reactive gliosis, involving astrocytes and microglia, is increasingly being recognised, not only as a pathological hallmark, but as a contributor to neurodegenerative disease and there is significant interest in identifying a metabolic signature that reflects gliosis *in vivo*^18,19^. We previously showed astrogliosis at 6 weeks-old in *Psmc1*^fl/fl^;*CaMKIIα-Cre* cortices by immunohistochemistry^11^. Here we extend our data to show reactive astrogliosis is evident from 3 weeks-old by glial fibrillary acidic protein (GFAP) immunostaining and immunoblotting, well before evidence of neurodegeneration (Fig. 3 and Supplementary Fig. 2)^52^. Quantitative immunoblotting analysis showed GFAP was significantly increased from 3 weeks-old in *Psmc1*^fl/fl^;*CaMKIIα-Cre* cortices, and increased from 1.97- to 19.54-fold between 3 and 6 weeks-old (Fig. 3B and Supplementary Fig. 7). Astrocyte activation was less dramatic in the hippocampus, increasing from 1.79- to 5.90-fold between 4 and 6 weeks-old (Fig. 3C and Supplementary Fig. 7).

**Figure 4:**
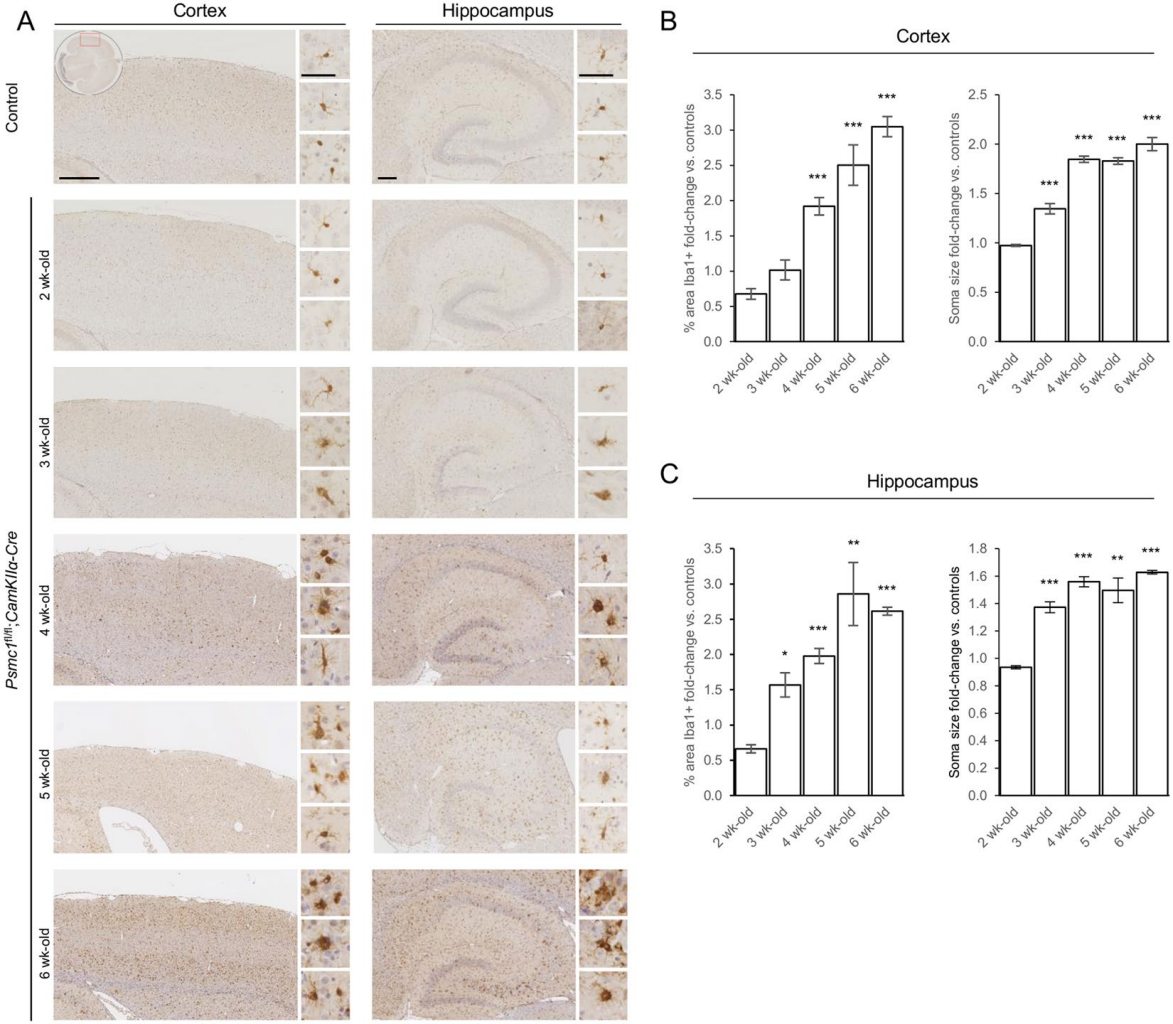
Microgliosis in *Psmc1*^fl/fl^;*CaMKIIα-Cre* forebrain. (**A**) Representative horizontal brain sections immunostained with Iba1. No significant differences were evident in control cortices; therefore the control image is representative of all ages. Insets show the region of cortex shown (LHS; oval) and higher magnification images of the pathology (RHS; white boxes). Scale bar = 500 (LHS; cortex) and 200 (RHS; hippocampus) µm. Quantitation of microglia in control and *Psmc1*^fl/fl^;*CaMKIIα-Cre* cortices (**B**) and hippocampi (**C**) using the percentage area stained by Iba1 and microglial soma size. Left and right cortices or hippocampi of n = 3 mice at each age were analysed; mean ± SEM. *p < 0.05, **p < 0.01 and ***p < 0.001 by unpaired Students *t*-test.

Interestingly, immunohistochemistry for the ionized calcium-binding adapter molecule 1 (Iba1) microglial marker showed reactive microgliosis was also an early feature in *Psmc1*^fl/fl^;*CaMKIIα-Cre* cortices (Fig. 4). Iba1 antibodies were not sensitive enough for immunoblotting at all ages, but semi-quantitative analysis of the immunostaining showed the percentage area of the cortex stained by Iba1 in *Psmc1*^fl/fl^;*CaMKIIα-Cre* mice was significantly increased from 4 weeks-old, and increased from 1.92- to 3.05-fold between 4 and 6 weeks-old (Fig. 4B). More important, quantitation of microglial soma size revealed a significant increase from 3 weeks-old in *Psmc1*^fl/fl^;*CaMKIIα-Cre* cortices, increasing from 1.35- to 2.00-fold at 6 weeks-old (Fig. 4B). Similar changes in microglia were observed in *Psmc1*^fl/fl^;*CaMKIIα-Cre* hippocampi (Fig. 4C). Changes in microglial soma size in *Psmc1*^fl/fl^;*CaMKIIα-Cre* mice are supported by Iba1 immunostaining showing perikaryal hypertrophy and amoeboid appearance with retracted processes indicative of an activated phenotype (Fig. 4A). Control microglia displayed a resting morphology, with smaller compact perikarya and many long thin ramified processes (Fig. 4A).

### Putative metabolic changes that track gliosis

**Figure 5:**
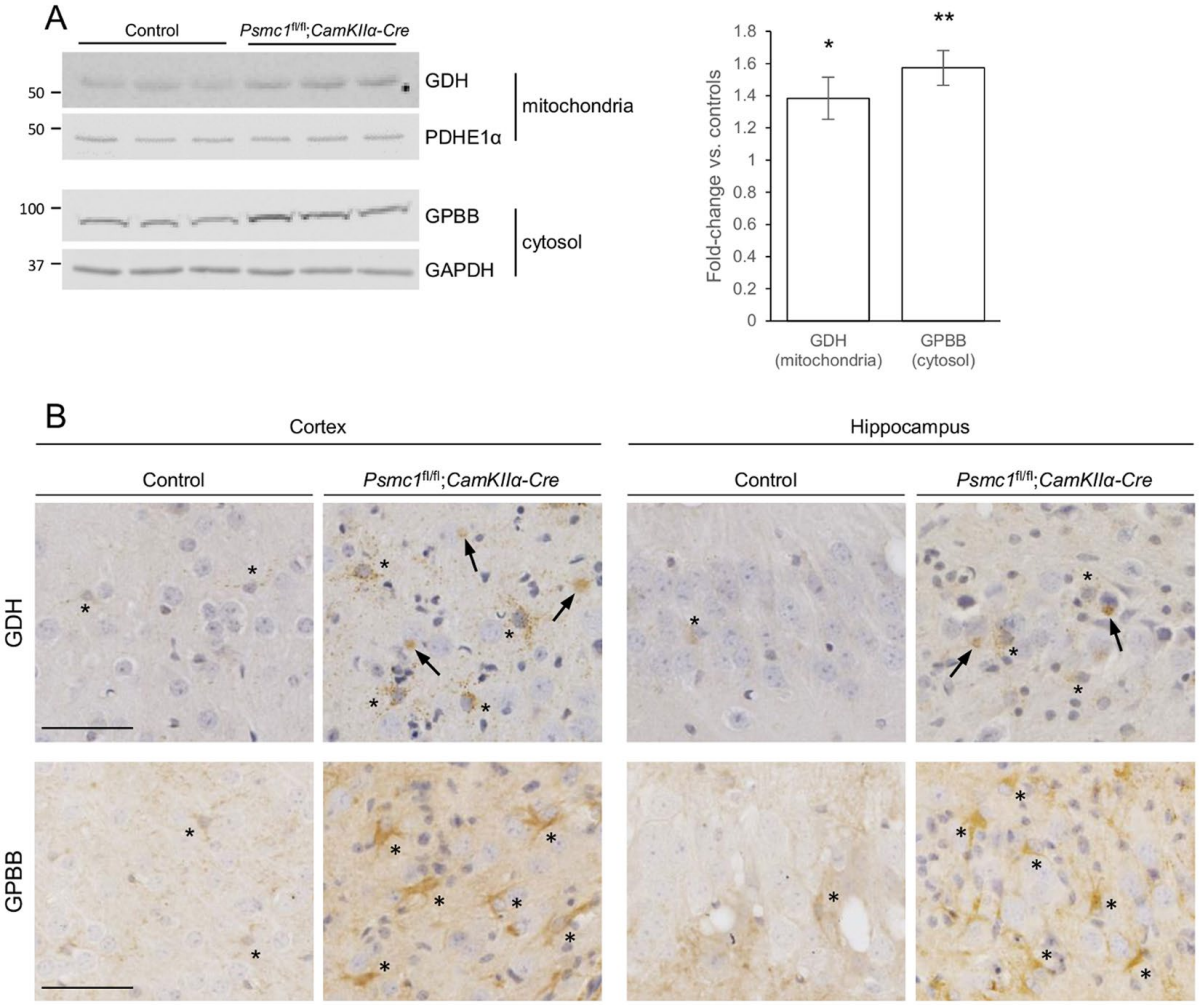
Increased GDH and GPBB in *Psmc1*^fl/fl^;*CaMKIIα-Cre* forebrain. (**A**) Representative immunoblots and quantification of GDH and GPBB in mitochondria and cytosol respectively purified from control and *Psmc1*^fl/fl^;*CaMKIIα-Cre* cortices at 6 weeks-old. Pyruvate dehydrogenase E1 component subunit alpha (PDHE1α; mitochondria) and glyceraldehyde 3-phosphate dehydrogenase (GAPDH; cytosol) were used as a loading controls. Images were cropped from those shown in Supplementary Figure 6. Mean ± SEM of n = 3 mice. *p < 0.05 and **p < 0.01 by unpaired Students *t*-test. (**B**) Immunostaining of cortical and hippocampal brain sections from control and *Psmc1*^fl/fl^;*CaMKIIα-Cre* mice at 6 weeks-old with GDH and GPBB. Asterisks and arrows indicate astrocytes and Lewy-like inclusions respectively. Scale bar = 50 µm.

Comparisons of the trajectories of metabolite and gliosis changes in *Psmc1*^fl/fl^;*CaMKIIα-Cre* cortices may inform on putative metabolite changes that track gliosis (Supplementary Fig. 10). The greatly increasing trajectory of GFAP in *Psmc1*^fl/fl^;*CaMKIIα-Cre* cortices may correlate with the significant changes in glutamate homeostasis, and possibly lactate (Figs 2 and 3). Interestingly, we support this by showing significantly increased levels of glutamate dehydrogenase (1.38-fold; GDH) and glycogen phosphorylase (1.57-fold; GPBB) in *Psmc1*^fl/fl^;*CaMKIIα-Cre* cortices *vs*. controls (Fig. 5A and Supplementary Fig. 8). Further, increased astrocytic GDH and GPBB immunostaining in *Psmc1*^fl/fl^;*CaMKIIα-Cre* cortical and hippocampal brain sections (Fig. 5B). In addition, GDH was associated with neuronal Lewy-like inclusions (Fig. 5B).

## Discussion

Metabolite profiling is a more recent addition to the ‘omics’ field and gives a snapshot of a tissue’s physiology that may better our understanding of the multiple features of neurodegeneration. Using 400 MHz *NMR* spectroscopy we have characterised the dynamic metabolic profile of cortex, hippocampus and cerebellum in *Psmc1*^fl/fl^;*CaMKIIα-Cre* mouse brain between 2 and 6 weeks-old accompanying neurodegenerative changes in this context. Early decreases in NAA and taurine, and increases in lactate and choline-containing compounds, were evident from 4 weeks-old in *Psmc1*^fl/fl^;*CaMKIIα-Cre* cortices. These metabolic changes were followed by decreased glutamine and increased glutamate from 5 weeks-old. A late increase in myo-inositol was evident at 6 weeks-old in *Psmc1*^fl/fl^;*CaMKIIα-Cre* cortices. Metabolic differences were corroborated in the hippocampus, but not observed in the cerebellum up to 6 weeks-old. Differential age trajectories of key metabolites reflect the dynamic forebrain metabolome of *Psmc1*^fl/fl^;*CaMKIIα-Cre* mice. We also reveal an early significant glial response to targeted neuronal dysfunction and by comparing significant metabolic changes with pathological and molecular alterations provide novel insights into the neuronal and glial responses to neuronal 26S proteasome dysfunction.

Interestingly, our study shows a significant decrease in cortical and hippocampal NAA levels in *Psmc1*^fl/fl^;*CaMKIIα-Cre* mice from 4 weeks-old before significant neuronal loss, indicating NAA may correlate with early neuronal dysfunction (Supplementary Fig. 10 A and data not shown)^11,52^. NAA is highly concentrated in neurons and as one of the most abundant metabolites in ^1^H *MRS* of the brain is employed as a non-invasive marker for neuronal health, providing a tool for diagnosis and evaluation of various neuropathologies in the clinical setting as well as the effects of potential neuroprotective therapies^29–31^. Numerous correlative clinical *MRS* studies and animal models have shown NAA reduction in neurodegenerative disease^32–35^.

The physiological functions of NAA, however, remain controversial. Substantial evidence couples NAA synthesis to energy production in neuronal mitochondria and decreased NAA may reflect primarily an impaired mitochondrial metabolism rather than neuronal loss, suggesting that reduced NAA could simultaneously represent both neuronal dysfunction as well as death^27–29^. Interestingly, we found a positive correlation between NAA and adenosine nucleotides in *Psmc1*^fl/fl^;*CaMKIIα-Cre* forebrain [Spearman correlation coefficient *r*_*μμ*_ = 0.60 (cortex) and 0.74 (hippocampus), *p* < 0.0001]. More importantly, we recently reported that *Psmc1*^fl/fl^;*CaMKIIα-Cre* cortical neurons show early mitochondrial changes, evidenced by paranuclear aggregation of mitochondria from 3 weeks-old^52^. These observations were associated with morphologically abnormal, fragmented mitochondria and furthermore, with decreased mitochondrial membrane potential and complex I activity^52^. Here we extend our previous mitochondrial analyses by quantifying the number of cytochrome oxidase IV (COXIV)-positive mitochondrial aggregates in *Psmc1*^fl/fl^;*CaMKIIα-Cre* cortical neurons between 2 and 6 weeks-old (Supplementary Fig. 9). We show that the number of paranuclear mitochondrial aggregates increases between 3 and 5 weeks-old, and then decreases at 6 weeks-old coinciding with increasing loss of neurons. The early significant decrease in NAA, therefore, may also be associated with changes in brain neuroenergetics, supporting the use of this metabolite to track neuronal health as well as death, underpinning clinical *MRS* studies in neurodegenerative disease (Supplementary Fig. 10 A and B). Increased lactate and decreased creatine also support impaired brain energy homeostasis in *Psmc1*^fl/fl^;*CaMKIIα-Cre* mice^61^. Given that the *Psmc1*^fl/fl^;*CaMKIIα-Cre* mouse model shows neuroinflammation and mitochondrial dysfunction accompanying neurodegeneration, features shared by multiple neurodegenerative diseases, our study may provide mechanistic insight and translation relevant to diverse diseases.

Glutamate homeostasis is significantly altered in *Psmc1*^fl/fl^;*CaMKIIα-Cre* cortices and may correlate with astrogliosis; glutamate is increased and glutamine decreased at 5 and 6 weeks-old (Supplementary Fig. 10 C and D). Most ^1^H *MRS*-based metabolomic studies report a decrease in the levels of glutamate, along with NAA, in experimental and clinical neurodegeneration^35^. Brain glutamate metabolism is complex and astrocytes play an integral role in the glutamate-glutamine cycle by removing glutamate from the synapse and amidating it to glutamine to be exported back to neurons, providing the precursor for the major excitatory and inhibitory transmitters glutamate and γ-aminobutyric acid^62^. This cycle is open, actively interfacing with other pathways, i.e. intermediary metabolism, and their biochemical specialisation allows astrocytes to dynamically adjust the formation of glutamine with the oxidation of glutamate for energy via deamination to α-ketoglutarate by GDH and entry into the tricarboxylic acid cycle (TCA)^63^. Increased astrocytic GDH in *Psmc1*^fl/fl^;*CaMKIIα-Cre* cortices suggests a shift in the metabolic fate of glutamate from conversion to glutamine towards oxidative metabolism that may be sustaining the energy demands of astrogliosis^64^. Interestingly, GDH was also associated with neuronal Lewy-like inclusions. It is possible that this is due to paranuclear aggregation of mitochondria in *Psmc1*^fl/fl^;*CaMKIIα-Cre* neurons^52^. However, GDH was recently shown to be important for oxidative metabolism of glutamine in neurons during elevated energy demand^65^. Therefore, increased neuronal metabolism of glutamine-derived carbon in the TCA cycle in an attempt to support respiration may contribute to the changes in glutamate homeostasis in *Psmc1*^fl/fl^;*CaMKIIα-Cre* cortices. We cannot exclude that glutamine is also exported out of the brain into the circulation to maintain nitrogen levels during amino acid oxidation.

It is possible that astrocytes respond adaptively to the bioenergetic challenges in *Psmc1*^fl/fl^;*CaMKIIα-Cre* neurons, leading to elevated lactate (Supplementary Fig. 10E). Intercellular metabolic coupling by the lactate shuttle may serve a protective function by ensuring a supply of substrates for activity in neurons, which convert lactate to pyruvate for oxidative metabolism. Some oxidised glutamate in astrocytes may form lactate by a partial pyruvate recycling pathway in *Psmc1*^fl/fl^;*CaMKIIα-Cre* cortices^66–68^. Importantly, elevated lactate in *Psmc1*^fl/fl^;*CaMKIIα-Cre* cortices may also be due to astrocytic utilisation of glucose and/or glycogen; supported by increased GPBB in *Psmc1*^fl/fl^;*CaMKIIα-Cre* astrocytes. Lactate may be transferred to neurons or used to support astrogliosis in this context. We note that lactate was significantly increased at 4 and 5 weeks-old, but decreased at 6 weeks-old in *Psmc1*^fl/fl^;*CaMKIIα-Cre* cortices. This is most likely explained by exhaustion of the relatively low astrocytic glycogen stores in brain caused by the continuing energetic demands in *Psmc1*^fl/fl^;*CaMKIIα-Cre* neurons and extensive astrogliosis^69,70^.

The myo-inositol response in our study is surprising given the early and extensive gliosis evident in *Psmc1*^fl/fl^;*CaMKIIα-Cre* cortices (Supplementary Fig. 10F-H). Activation of glia has been associated with several metabolites using ^1^H *MRS* in patients and models, but changes in myo-inositol, which is mostly located in glial cells, are expected to represent the extent of gliosis^36–39^. However, more recent studies have not found an association between neuropathologic measures reflecting astrocyte or microglia activation and myo-inositol levels, suggesting that myo-inositol may not be sensitive to glial activation and mainly reflects glial cell density in line with its elevation in gliomas^39,41,42^. Furthermore, low baseline cortical myo-inositol levels and potential interspecies differences may contribute to the apparent lower sensitivity of myo-insoitol in mouse models compared to humans. It is worth noting that glycine significantly increased from 5 weeks-old and with age in both *Psmc1*^fl/fl^;*CaMKIIα-Cre* cortices and hippocampi, following the trajectory of GFAP (Supplementary Fig. 10I). Glycine and myo-inositol can easily be differentiated by *in vitro* ^1^H *NMR*, allowing quantitation of both metabolites here^45^. However, these metabolites cannot be reliably separated using standard clinical ^1^H *MRS* making it possible that glycine changes contribute to the myo-inositol-attributed gliosis signal at 3.5–3.6 ppm in humans^36,37^. Taken together, the use of myo-inositol as a gliosis marker may have limited sensitivity. Specificity and interspecies differences still require clarification, highlighting the need for identification of additional metabolic neuroinflammation markers.

With the exception of glutamine, most amino acids were more abundant in *Psmc1*^fl/fl^;*CaMKIIα-Cre* compared to control cortices. The accumulation of proteasome substrates as a consequence of proteasome inhibition may limit critical amino acids for protein synthesis and the changes in amino acid homeostasis in *Psmc1*^fl/fl^;*CaMKIIα-Cre* mice may reflect a compensatory increase in amino acid supply across the blood brain barrier in an attempt to rescue the neurons^71^. Amino acids cannot be provided by autophagy in this context because continued 26S proteasome dysfunction impairs autophagic degradation^52^. An interesting study showed that amino acid supplementation could rescue cell death following proteasome inhibition in yeast, mammalian cells and flies^72^.

The significant increase in choline-containing compounds in *Psmc1*^fl/fl^;*CaMKIIα-Cre* cortices may be an indicator of the breakdown of membrane phospholipids such as phosphatidylcholine (PtdCho). PtdCho hydrolysis by phospholipase A_2_ (PLA_2_) produces *lyso*-PtdCho, GPCh, phosphocholine and free choline. We previously showed PLA_2_ activity is increased in *Psmc1*^fl/fl^*;CaMKIIα-Cre* cortices, some of which is associated with the bifunctional enzyme peroxiredoxin 6, but other phospholipases also contribute to this activity^73^. Changes in choline have been inconsistently reported in experimental and clinical research, but several studies suggest elevated choline may be associated with the breakdown of neurons during the progression of neurodegenerative disease or membrane turnover associated with fibrillary gliosis, supporting our observations^38,41,74–76^.

Taurine levels were significantly decreased in *Psmc1*^fl/fl^*;CaMKIIα-Cre* cortices from 4 weeks-old. Taurine has been implicated in diverse functions in brain, but one likely rational is its role as an osmolyte, and changes in its concentration may indicate imbalanced ion and water homeostasis in *Psmc1*^fl/fl^*;CaMKIIα-Cre* cortex^29,46^. A recent study showed an inverse correlation between astrocytic activity and taurine in a mouse model of Alzheimer’s disease, supporting evidence that taurine may be an anti-inflammatory molecule and non-invasive marker of neuroinflammation in the brain^77–79^. Interestingly, the overall trajectory of microglial changes in *Psmc1*^fl/fl^*;CaMKIIα-Cre* cortices may be inversely associated with the trajectory of taurine, further supporting the connection to neuroinflammation of this metabolite (Supplementary Fig. 10 J and K)^77–79^.

Future studies using pharmacological or genetic approaches to target astroglia and microglia with comparable metabolomics will be of great interest to enlighten further which metabolites best represent neuroinflammation. Taken together, our study contributes to limited knowledge of metabolite changes during significant neuroinflammation triggered as a consequence of neuronal dysfunction.

## Electronic supplementary material

Supplementary Information
