# Supplementary material for: Dynamic metabolic patterns tracking neurodegeneration and gliosis following 26S proteasome dysfunction in mouse forebrain neurons
Source: Sci Rep. 2018 Mar 19;8:4833. doi: 10.1038/s41598-018-23155-2 (PMC5859111; doi:10.1038/s41598-018-23155-2)
Supplement: Supplementary file 1 — Supplementary Information [file 41598_2018_23155_MOESM1_ESM.pdf]

Dynamic metabolic patterns tracking neurodegeneration and gliosis following 26S proteasome dysfunction in mouse forebrain neurons

Philippine C. Geiszler [1,2], Aslihan Ugun-Klusek [3], Karen Lawler [4], Marie-Christine Pardon [4], Ding Yuchun [5], Li Bai [6], Clare A. Daykin [2,7], Dorothee P. Auer [1,8], Lynn Bedford [4]

Supplementary Materials and Methods

Multivariate Statistical Analysis. Partial least squares (PLS) analysis - the regression variant of principal component analysis (PCA) - was applied to relate the spectral data ('X'-block) to mouse age ('Y'-block); and projection to latent structures by partial least squares discriminant analysis (PLS-DA) was deployed to define distinct group clusters. PLS-DA is the classification variant of PCA. The spectral data (`X'-block) was related to 2 categorical groups (*e.g. Pmsc1*$^{fl/fl}$;*CaMKIIα-Cre* and control; `Y'-block). R^2X*(cum)*, R^2Y*(cum)* and Q^2*(cum)* values calculated by PCA, PLS and PLS-DA algorithms inform about the robustness of X and Y's relationship and/or X's predictability of Y; with Q^2*(cum)* - calculated by a cross-validation strategy – over 0.7 being regarded as an indicator of a robust model (max. = 1) [1].

The PLS-DA results were further tested for their robustness through manual block and venetian blinds validation strategies using samples of the present (validation set) and the pilot (external test set) study. These validation sets provide a root mean square error of estimation/prediction (RMSEE/P), indicating the fit of the observed scores to the scores estimated by the PLS-DA model. Whilst RMSEE and RMSEP are displayed in original units (e.g. mouse age; days, PLS; unit-less when differentiating between control and *Pmsc1*$^{fl/fl}$;*CaMKIIα-Cre* mice), the regression coefficient r_m is expressed as a fraction of 1; with values over 0.8 showing a strong correlation between estimated and observed scores.

Supplementary Tables

Supplementary Table 1. Number of mice at each age used in *NMR* spectroscopy.

	**2 weeks-old**	**3 weeks-old**	**4 weeks-old**	**5 weeks-old**	**6 weeks-old**
Control	10	12	13	11	12
Psmc1$^{fl/fl}$;*CaMKIIα-Cre*	8	11	11	10	11

Supplementary Table 2. List of metabolites that changed significantly and consistently with mouse age between 2 and 6 weeks-old indicated by the Spearman correlation coefficient r_u. ***p*** values indicate maturation differences with respect to genotype. The difference between the age-related regression slopes of control and *Psmc1*$^{fl/fl}$;*CaMKIIα-Cre* was calculated for each brain region using web-based software [2]. NAA: *N*-acetyl aspartate; GABA: γ-aminobutyric acid; Cho: choline-containing compounds; *ns* = $p > 0.002$; **red bold** $r_u \geq 0.90$; **blue bold** $r_u \geq 0.8$; **black bold** $r_u \geq 0.60$.

METABOLITE	CORTEX			HIPPOCAMPUS			CEREBELLUM		
	Control	*Psmc1*$^{fl/fl}$; *CaMKIIα-Cre*	***p<***	Control	*Psmc1*$^{fl/fl}$; *CaMKIIα-Cre*	***p<***	Control	*Psmc1*$^{fl/fl}$; *CaMKIIα-Cre*	***p<***
Myo-inositol	**0.88**	**0.91**	*ns*	**0.71**	**0.93**	*0.001*	0.52	**0.78**	*ns*
Taurine	**-0.70**	**-0.83**	*ns*	**-0.75**	**-0.77**	*ns*	**-0.77**	**-0.81**	*ns*
NAA	-0.26	**-0.79**	*0.001*	**-0.71**	**-0.66**	*ns*	-0.38	-0.19	*ns*
Glutamine	0.06	**-0.63**	*0.001*	-0.04	**-0.69**	*0.001*	-0.44	-0.23	*ns*
Glycine	-0.08	**0.69**	*0.001*	0.24	**0.80**	*0.001*	-0.29	-0.16	*ns*
Glycerol	-0.08	**0.67**	*0.001*	-0.13	**0.67**	*0.001*	0.18	0.06	*ns*
GABA	**0.87**	**0.61**	*ns*	0.31	**0.62**	*ns*	**0.76**	**0.84**	*ns*
Cho	0.53	0.54	*ns*	0.21	0.41	*ns*	-0.46	-0.59	*ns*
Acetate	**0.62**	0.47	*ns*	**0.66**	0.12	*0.001*	0.46	0.53	*ns*
Serine	-0.16	-0.25	*ns*	-0.46	0.46	*0.001*	**-0.65**	**-0.71**	*ns*
Uracil	-0.14	-0.24	*ns*	-0.07	-0.19	*ns*	**-0.75**	**-0.82**	*ns*

Supplementary Table 3. Robustness of multivariate models showing the metabolic effects of maturation in cortex (CTX), hippocampus (HC) and cerebellum (CB) of control and *Psmc1*$^{fl/fl}$;*CaMKIIα-Cre* mice. Validation and internal test sets were used to validate the models and all models were based on two latent variables. Reported errors are standard deviations.

**GROUP**	**TRAINING**					**VALIDATION**	**INTERNAL TEST**	
	R^2X*(cum)*	R^2Y*(cum)*	Q^2*(cum)*	RMSEE [days]	r_m	RMSEP [d]	RMSEP [days]	r_m
**CTX:** control	0.70±0.13	0.85±0.03	0.78±0.05	3.85±0.77	0.92±0.02	8.66±5.93	4.65±0.65	0.87±0.06
**CTX:** *Psmc1*$^{fl/fl}$; *CaMKIIα-Cre*	0.82±0.03	0.85±0.01	0.94±0.02	2.12±0.17	0.98±0.01	4.05±2.24	3.02±0.09	0.95±0.04
**HC:** control	0.61±0.09	0.82±0.07	0.72±0.15	4.25±0.76	0.91±0.04	6.50±2.92	5.30±0.27	0.81±0.06
**HC:** *Psmc1*$^{fl/fl}$; *CaMKIIα-Cre*	0.67±0.05	0.96±0.01	0.95±0.02	1.86±0.21	0.98±0.01	3.37±1.36	2.58±0.58	0.97±0.03
**CB:** control	0.60±0.10	0.85±0.05	0.77±0.08	3.79±0.57	0.92±0.03	7.46±4.31	4.21±0.95	0.91±0.02
**CB:** *Psmc1*$^{fl/fl}$; *CaMKIIα-Cre*	0.72±0.08	0.88±0.03	0.82±0.05	3.44±0.36	0.94±0.02	7.43±4.35	6.59±0.43	0.75±0.06

Supplementary Table 4. Robustness of multivariate models showing the metabolic effects of genotype in hippocampus (HC) and cortex (CTX) of 4, 5 and 6 week-old (wk) control and *Psmc1*$^{fl/fl}$*;CaMKIIα-Cre* mice. Training sets were used to build the models and averages are reported here. Validation and independent external test sets were used to validate the models. Reported errors are standard deviations.

**GROUP**		**TRAINING**			
	LV	R^2X*(cum)*	R^2Y*(cum)*	Q^2*(cum)*	RMSEE
HC: 4 wk	1	0.36 ± 0.07	0.79 ± 0.04	0.72 ± 0.15	0.24 ± 0.02
HC: 5 wk	1	0.42 ± 0.01	0.96 ± 0.00	0.93 ± 0.01	0.10 ± 0.01
HC: 6 wk	2	0.67 ± 0.06	0.96 ± 0.00	0.95 ± 0.00	0.07 ± 0.01
CTX: 4 wk	3	0.86 ± 0.03	0.98 ± 0.02	0.94 ± 0.01	0.09 ± 0.02
CTX: 5 wk	2	0.85 ± 0.03	0.96 ± 0.02	0.98 ± 0.01	0.08 ± 0.01
CTX: 6 wk	2	0.85 ± 0.02	0.99 ± 0.00	0.99 ± 0.01	0.05 ± 0.01

	**VALIDATION**		**EXTERNAL TEST**	
	RMSEP	misclassified samples	RMSEP	misclassified samples
HC: 4 wk	0.27 ± 0.05	2 out of 8	0.277 ± 0.01	1.3 out of 6
HC: 5 wk	0.14 ± 0.03	0.3 out of 7	–	–
HC: 6 wk	0.11 ± 0.02	1 out of 7	–	–
CTX: 4 wk	0.13 ± 0.97	0 out of 8	0.14 ± 0.02	0 out of 6
CTX: 5 wk	0.09 ± 0.03	0 out of 6	–	–
CTX: 6 wk	0.06 ± 0.01	0 out of 8	–	–

Supplementary Figures

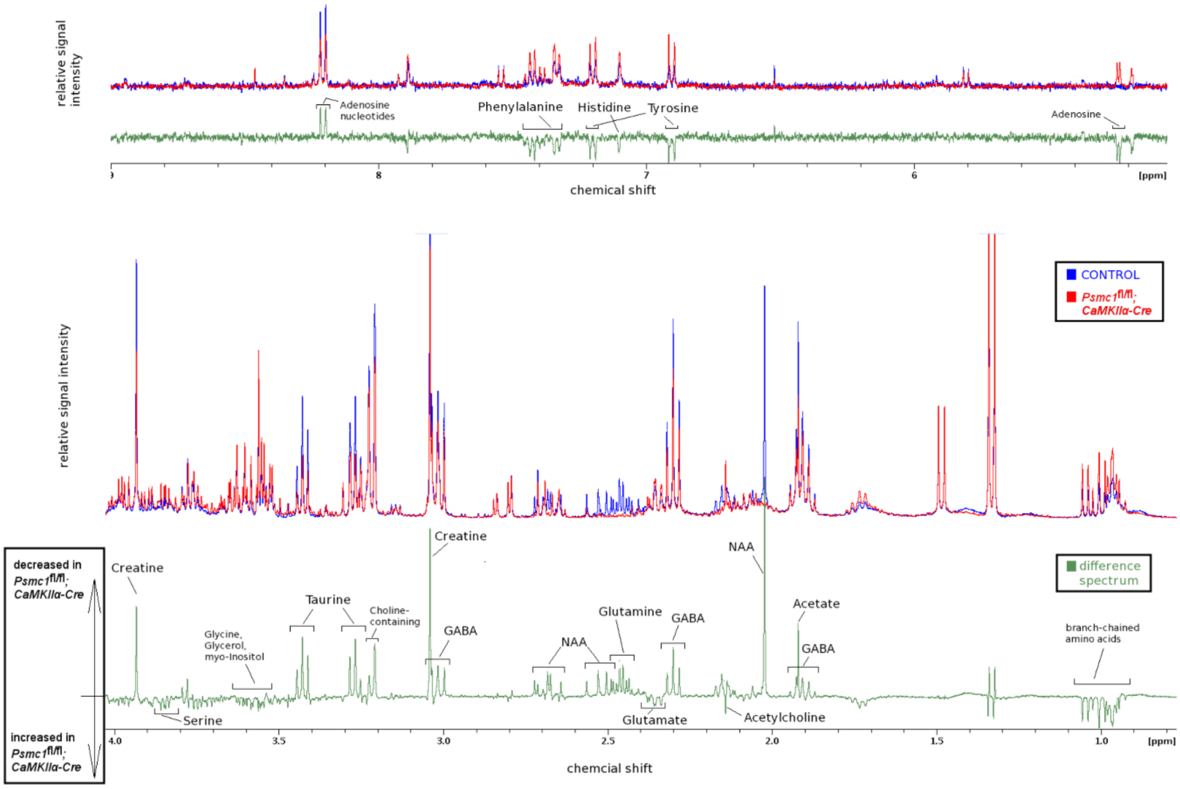

Supplementary Figure 1. Representative high resolution 1D ^{1}H *NMR* spectra of a 6 week-old control (blue) and *Psmc1*$^{fl/fl}$*;CaMKIIα-Cre* (red) mouse cortex acquired at 9.4 Tesla (400 MHz). Tentative peak assignments are indicated where differences between the two genotypes are visible in the spectra. Arrows next to the difference spectrum (green) indicate the direction of change in *Psmc1*$^{fl/fl}$*;CaMKIIα-Cre* mice.

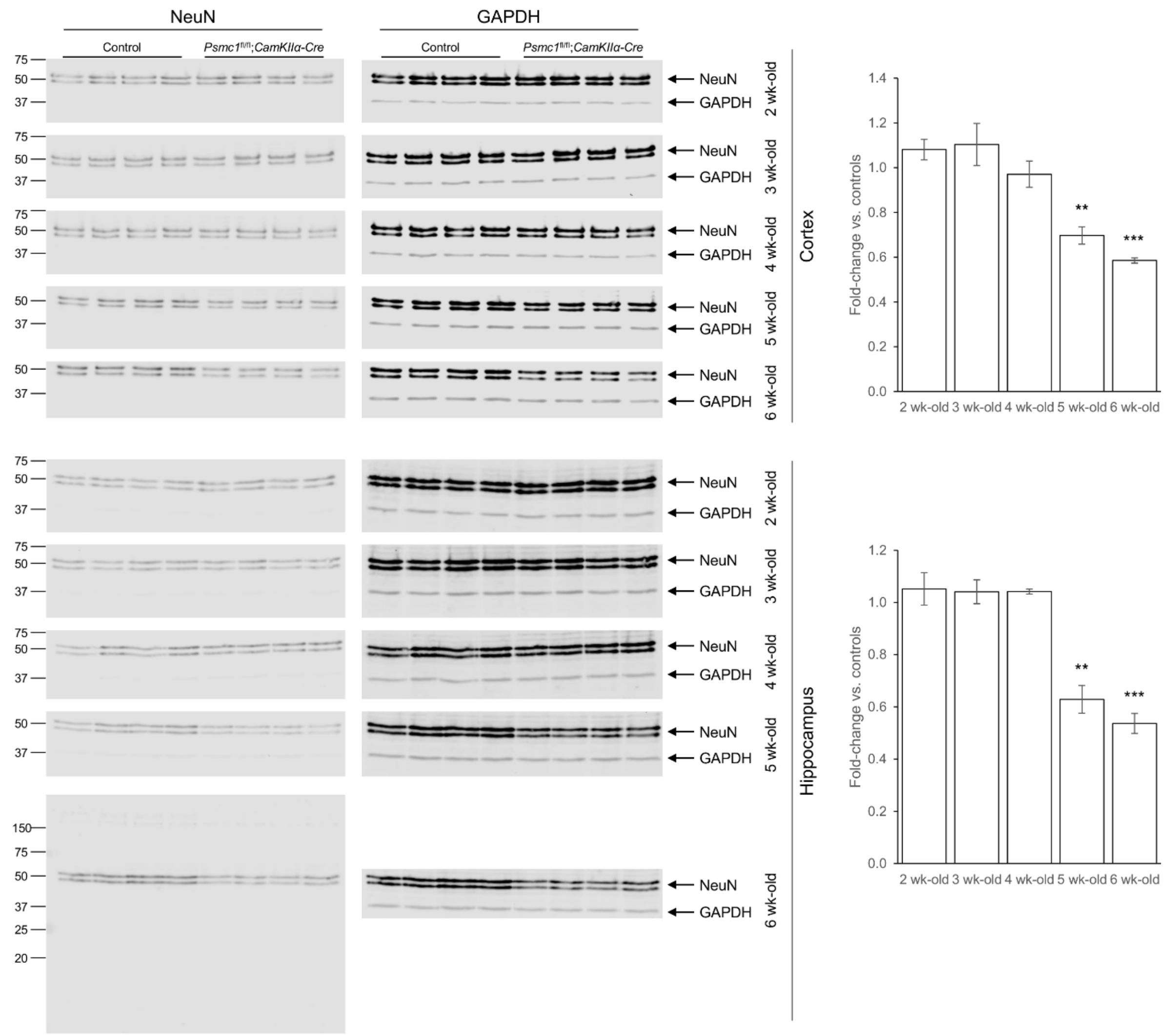

Supplementary Figure 2. Immunoblots and quantification of NeuN in control and *Psmc1*$^{fl/fl}$;*CaMKIIα-Cre* cortices (top) and hippocampi (bottom). Glyceraldehyde 3-phosphate dehydrogenase (GAPDH) was used as a loading control at each age for quantification. The membrane was cut between approximately 25 and 75 kDa before immunoblotting; a full blot was used for NeuN 6 week-old hippocampus to show there are no non-specific bands. NeuN (LHS) immunoblotting was carried out first and then GAPDH (RHS) on the same membrane; NeuN signal is still detected following immunoblotting for GAPDH. Mean ± SEM of n = 4 mice. **$p < 0.01$ and ***$p < 0.001$ by unpaired Students *t*-test.

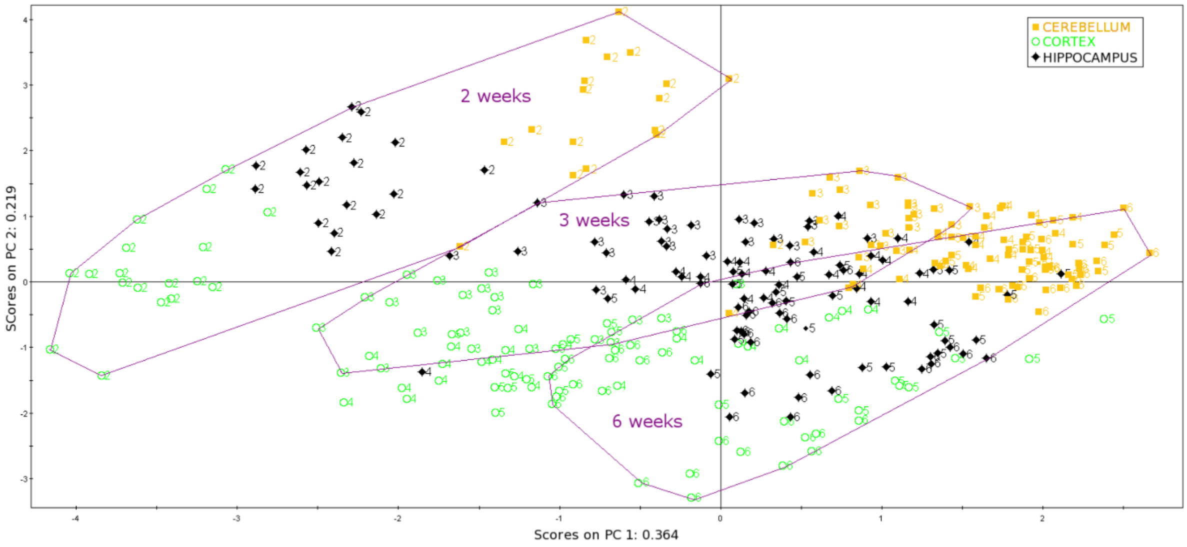

Supplementary Figure 3. PCA scores plot demonstrating significant metabolic differences between brain regions and age groups. Numerical annotation indicates the mouse age in weeks; 2, 3 and 6 week-old age groups have been highlighted by manually joining the outer points at each age. 2 PCs; R2X*(cum)* = 0.703.

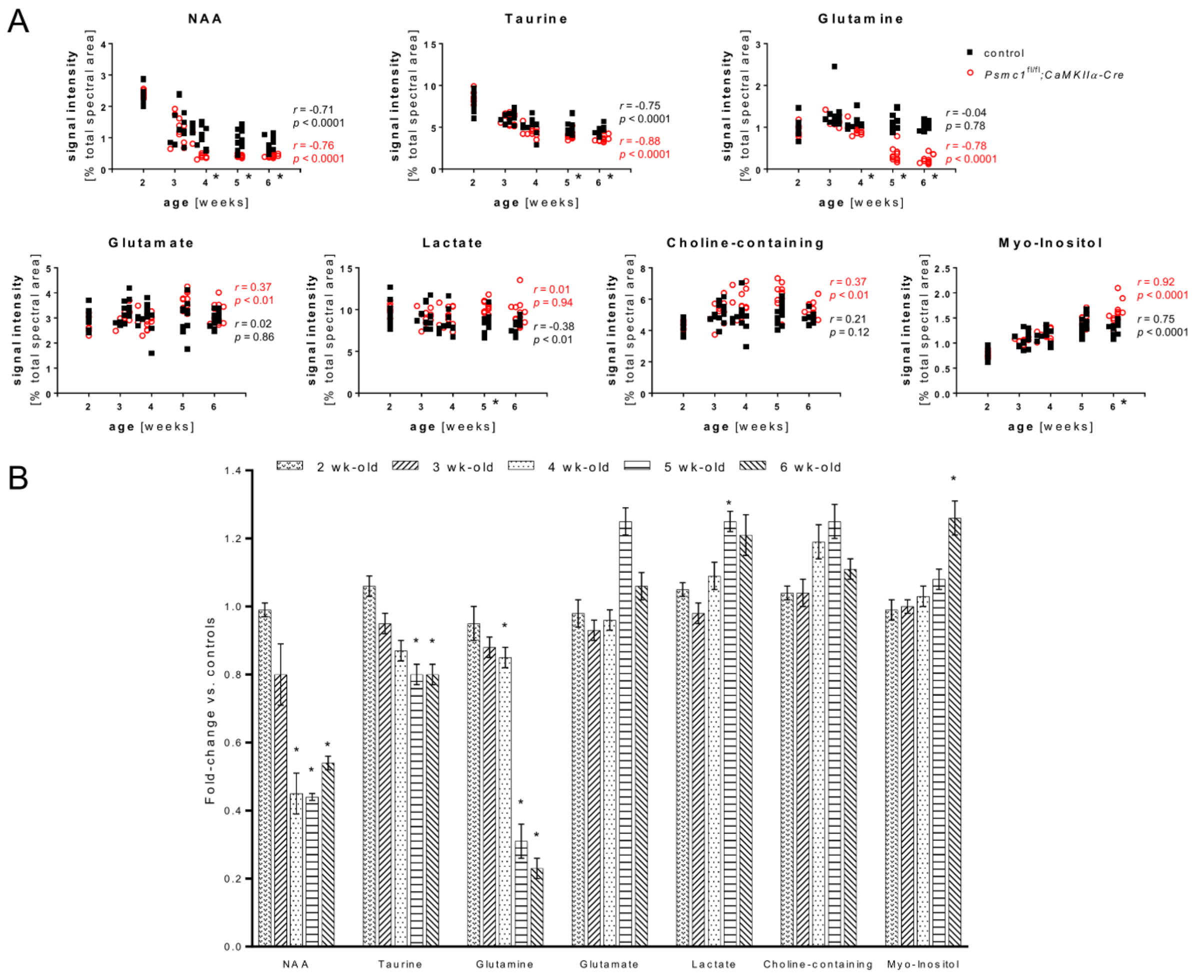

Supplementary Figure 4. Differences in metabolite concentration between control and *Psmc1*$^{fl/fl}$*;CaMKIIα-Cre* hippocampi. (A) Correlation plots between metabolite concentration and age expressed as % signal intensity relative to the total spectral area for control (black) and *Psmc1*$^{fl/fl}$*;CaMKIIα-Cre* (red) hippocampi. (B) Differences in metabolite concentration in *Psmc1*$^{fl/fl}$*;CaMKIIα-Cre* mice expressed as fold-change *vs*. controls. Mean ± SEM. For A and B; n = Supplementary Table 1, **p* < 0.002 by Man-Whitney U test.

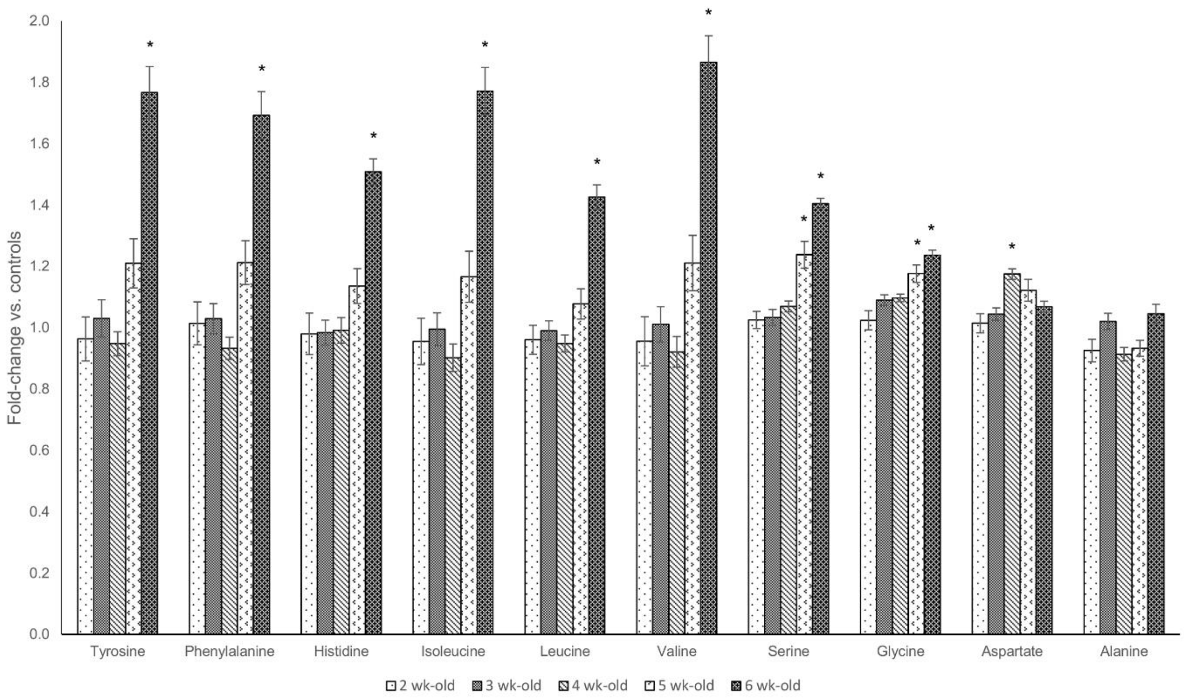

Supplementary Figure 5. Differences in amino acids between control and *Psmc1*$^{fl/fl}$*;CaMKIIα-Cre* cortices. Differences in metabolite concentration in *Psmc1*$^{fl/fl}$*;CaMKIIα-Cre* mice expressed as fold-change *vs*. controls. Mean ± SEM of n = Supplementary Table 1; *p < 0.002 by Man-Whitney U test.

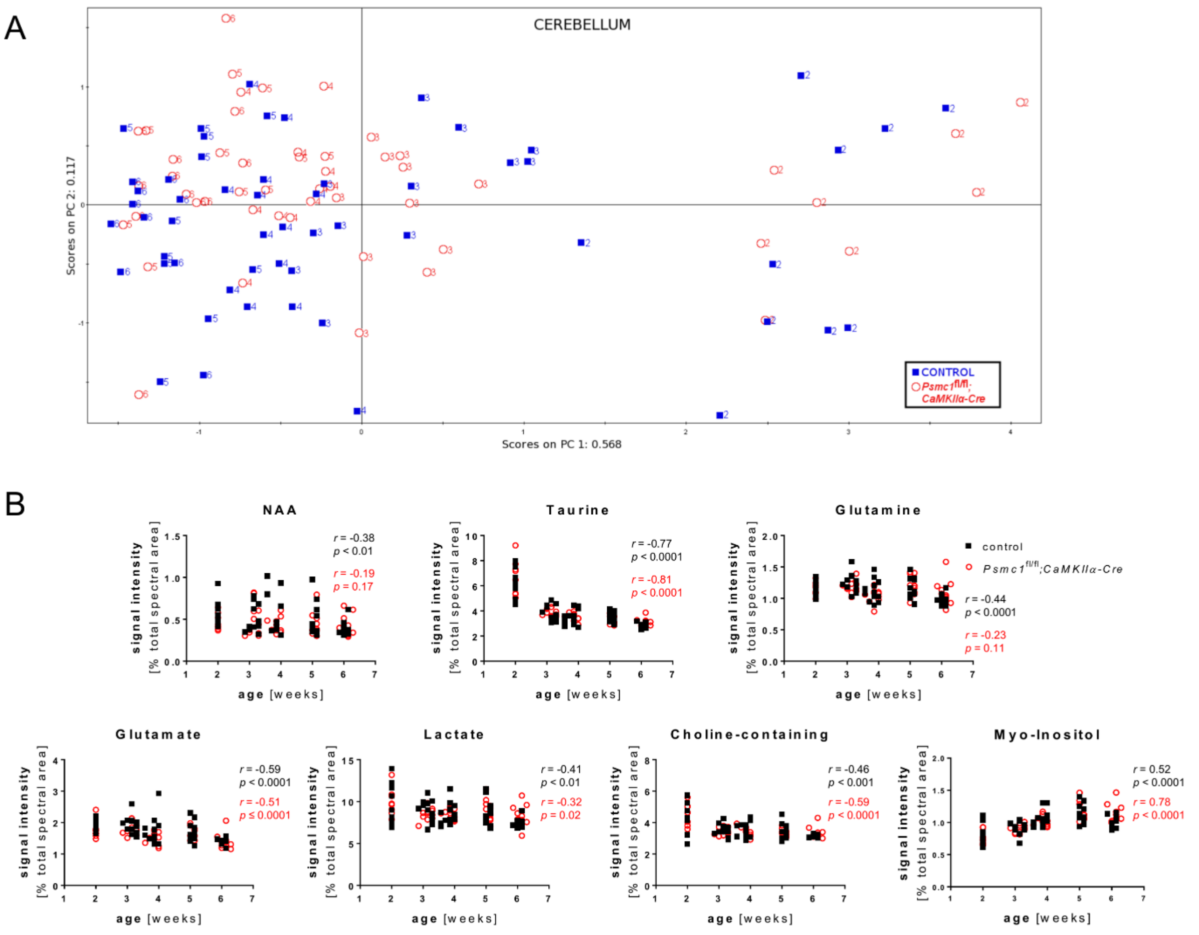

Supplementary Figure 6. (A) PCA scores plot of cerebellum extracts displaying metabolic variations between samples from 2-6 weeks-old. There was no indication of a metabolic maturation difference between control (blue square) and *Psmc1*$^{fl/fl}$;*CaMKIIα-Cre* (red circle) mice. Numerical annotation indicates the mouse age in weeks. 2 PCs; $R^2X(cum)$ = 0.686. (B) Correlation plots between metabolite concentration and age expressed as % signal intensity relative to the total spectral area for control (black) and *Psmc1*$^{fl/fl}$;*CaMKIIα-Cre* (red) cerebellums.

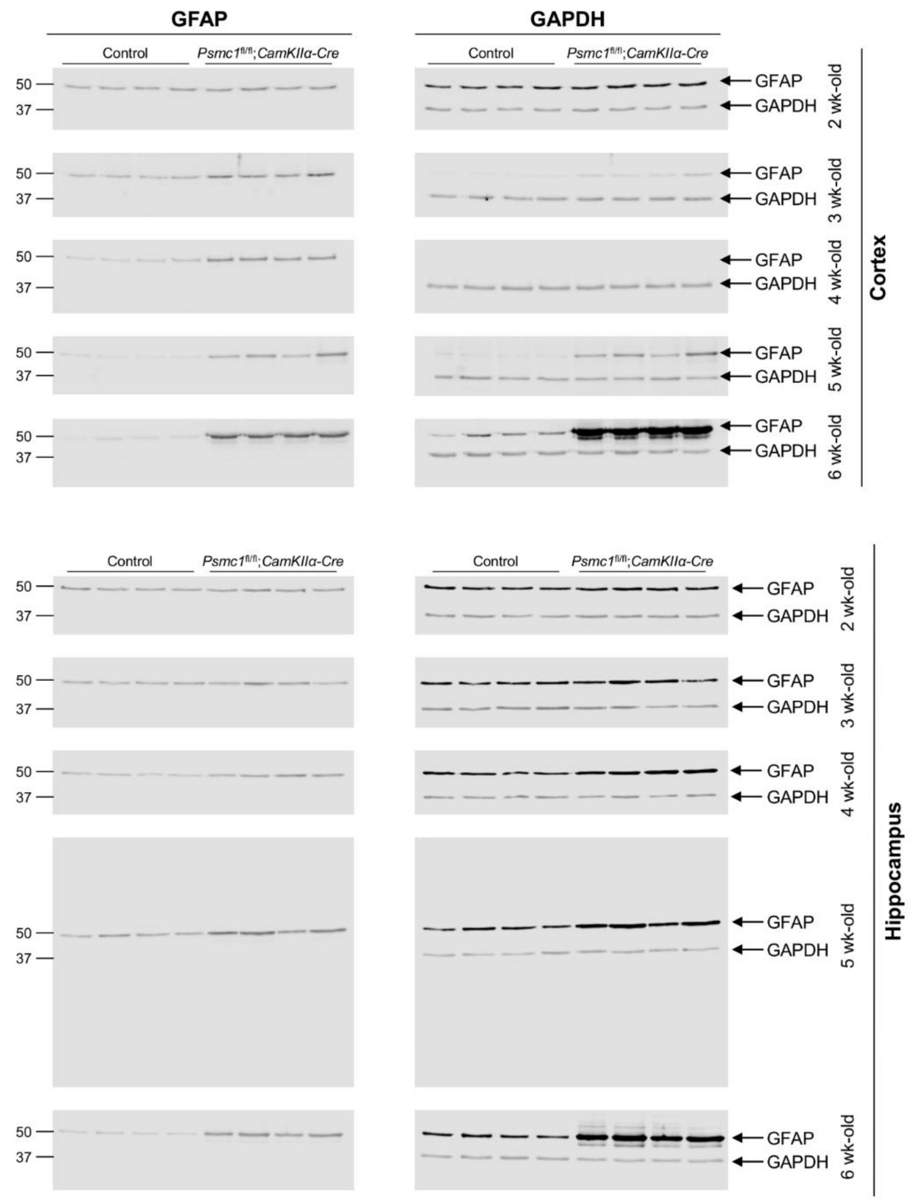

Supplementary Figure 7. Immunoblots of GFAP and GAPDH in control and *Psmc1*$^{fl/fl}$;*CaMKIIα-Cre* cortices (top) and hippocampi (bottom) shown in Figure 3. The membrane was cut between approximately 30 and 60 kDa before immunoblotting; a full blot was used for GFAP and GAPDH 5 week-old hippocampus to show there are no non-specific bands. GFAP (LHS) immunoblotting was carried out first and then GAPDH (RHS) on the same membrane; GFAP signal may still be detected following immunoblotting for GAPDH. The images shown in Figure 3 are cropped from those shown here. The GAPDH at 4 weeks-old is shown in Figure 3.

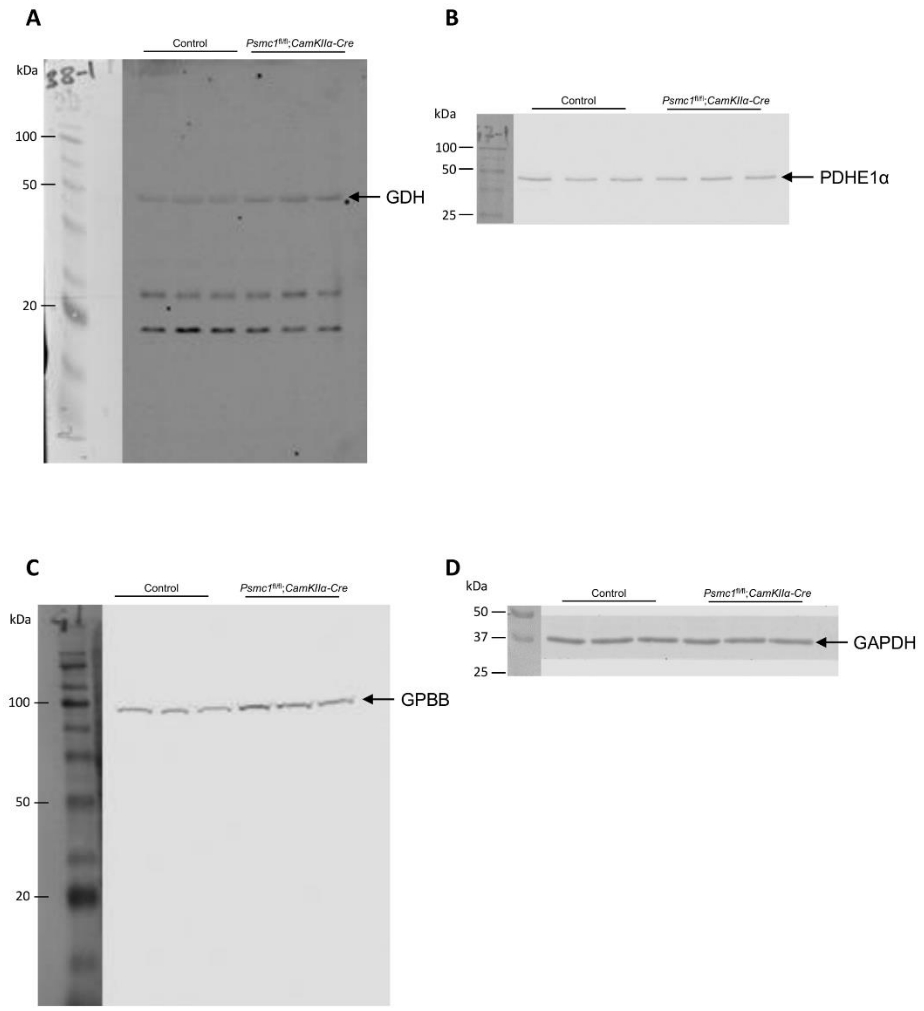

Supplementary Figure 8. (A and B) Immunoblots of GDH (A) and PDHE1α (B) in mitochondria purified from control and *Psmc1*$^{fl/fl}$;*CaMKIIα-Cre* cortices at 6 weeks-old shown in Figure 5. The full immunoblot for GDH is shown (A). The same samples were loaded on a different gel for PDHE1α (B) and the membrane was cut at the bottom before immunoblotting between the 20 and 25 kDa markers. (C and D) Immunoblots of GPBB (C) and GAPDH (D) in cytosol purified from control and *Psmc1*$^{fl/fl}$;*CaMKIIα-Cre* cortices at 6 weeks-old shown in Figure 5. The full immunoblot for GPBB is shown (C). The same samples were loaded on a different gel for GAPDH (D) and the membrane was cut before immunoblotting between the 25 and 50 kDa markers. The images shown in Figure 5 are cropped from those shown here.

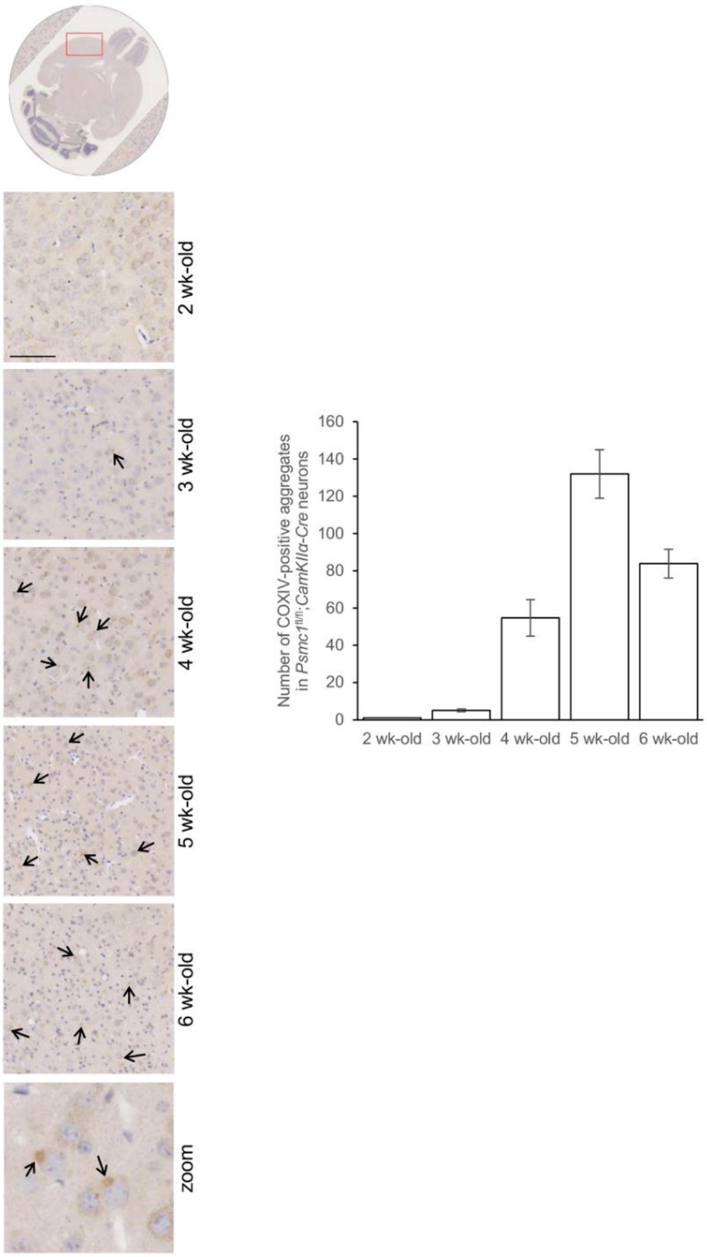

Supplementary Figure 9. Quantification of the number of COXIV-positive mitochondrial aggregates in *Psmc1*$^{fl/fl}$;*CaMKIIα-Cre* neurons between 2 and 6 weeks-old. Representative horizontal brain sections immunostained with cytochrome oxidase IV (COXIV). Mitochondrial aggregates are not found in control neurons; therefore control images are not shown. Top image shows the region of cortex examined and bottom image a higher magnification of the neuropathological finding (arrows). Scale bar = 100 µm. Left and right cortices of n = 3 mice at each age were analysed; mean ± SEM.

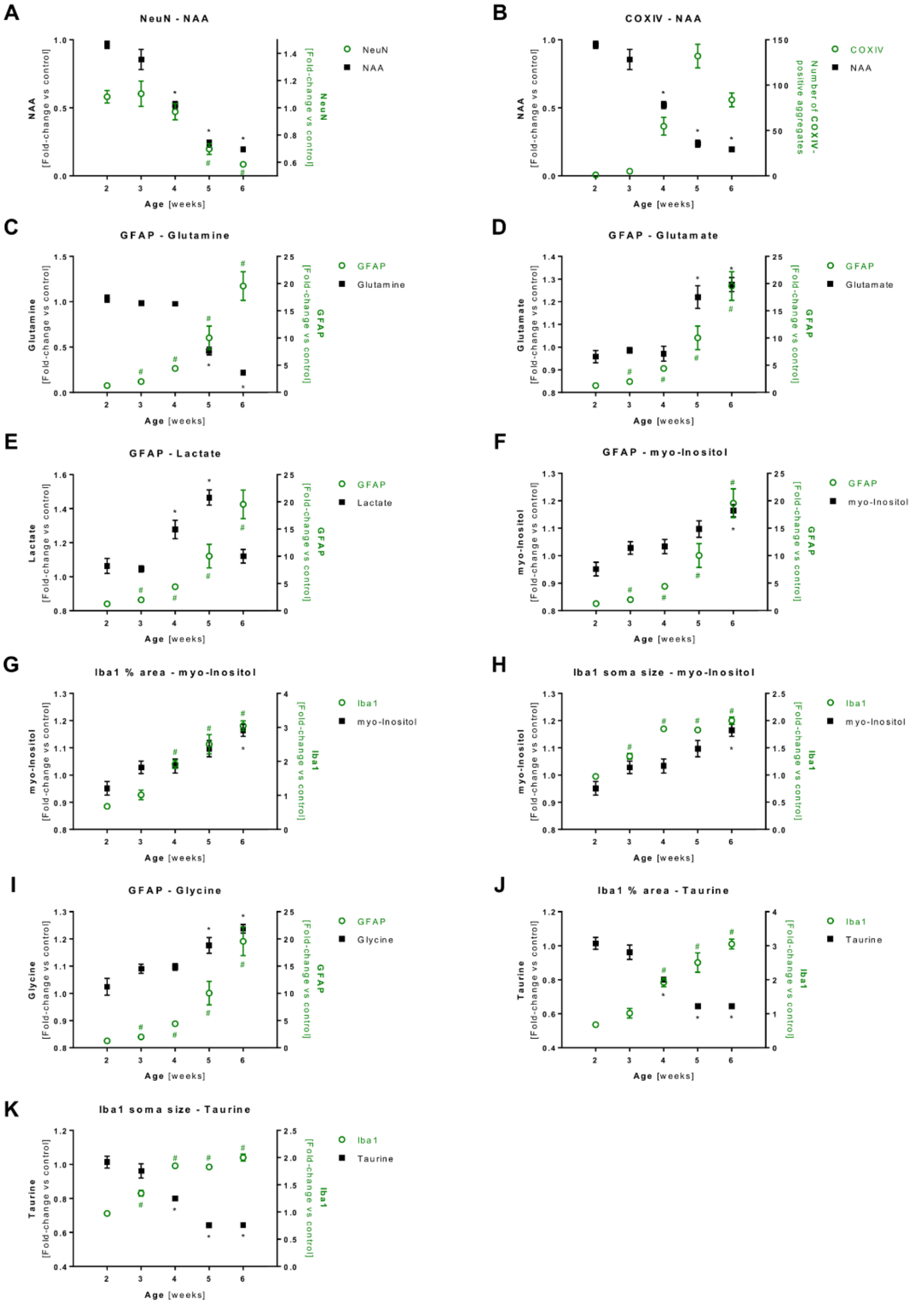

Supplementary Figure 10. Timelines depicting progression of neuropathological findings (green) in relationship to major metabolites (black) in *Psmc1*$^{fl/fl}$;*CaMKIIα-Cre* cortices. # and * represent significant differences in *Psmc1*$^{fl/fl}$;*CaMKIIα-Cre* vs. control cortices for neuropathological findings or metabolites respectively from Figures 2-4.

References

1 Salek, R. M. *et al.* A metabolomic study of brain tissues from aged mice with low expression of the vesicular monoamine transporter 2 (VMAT2) gene. *Neurochemical Research* **33**, 292-300, doi:DOI 10.1007/s11064-007-9542-3 (2008).

2 Maier, A. H., Florian; Bocklet, Tobias; Nöth, Elmar; Stelzle, Florian; Nkenke, Emeka; Schuster, Maria. (2009) *Correlation Comparison*, <http://peaks.informatik.uni-erlangen.de/cgi-bin/usignificance.cgi>.
